# Folate-targeted gold nanoparticles for doxorubicin delivery in tumor spheroids

**DOI:** 10.1080/10717544.2025.2607390

**Published:** 2025-12-30

**Authors:** Raffaella Daniele, Agnese Fragassi, Cristiano Pesce, Francesco Tognetti, Marco Verona, Giovanni Marzaro, Stefano Salmaso, Paolo Caliceti

**Affiliations:** a Department of Pharmaceutical and Pharmacological Sciences, University of Padova, Padova, Italy; b Department of Diagnostics and Public Health, University of Verona, Verona, Italy

**Keywords:** Nanoparticle-drug conjugates, gold nanoparticles, controlled drug release, anticancer drug delivery, pH responsive systems

## Abstract

Targeted drug delivery systems represent a promising strategy for enhancing the efficacy and specificity of cancer therapy. In this study, 35 nm folate-targeted gold nanoparticles are presented as nanoparticle–drug conjugates obtained by anchoring on their surface lipoyl terminating doxorubicin prodrug (proDoxo) releasable at the endolysosomal acidic pH to prevent off-site toxic effects. Colloidal stable nanoparticles with a density of proDoxo up to 1000 molecules/particle and 2 kDa mPEG-SH coating were obtained. At pH 5, Doxo was completely released from the nanoparticles in 5 days while only 13% was released over the same period at pH 7.4. The nanoparticle decoration with folic acid as a targeting agent bestowed nanosystems with selective drug delivery to folate receptor (FR)-overexpressing cancer cells and controlled intracellular release. This led to enhanced cancer cell killing by folated nanoparticles compared to their nontargeted counterparts. Moreover, folated nanoparticles were found to distribute more homogeneously inside KB^FR+^ cancer cell spheroids than non-targeted nanoparticles, resulting in higher spheroid volume reduction.

## Graphical abstract

**Figure:**
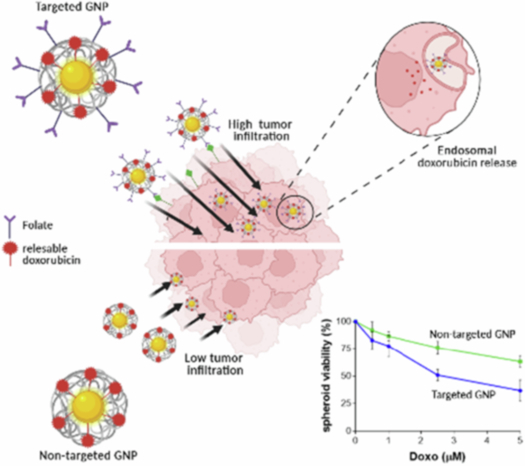

Folate-targeted gold nanoparticles decorated with a pH-releasable doxorubicin prodrug achieve selective and controlled drug release and enhanced folate receptor (FR)-positive cancer cell killing. With up to 1000 doxorubicin/particle, PEG coatings ensured remarkable colloidal stability. Folate decoration promotes homogeneous distribution into FR-positive KB cell spheroids, yielding superior therapeutic efficacy over non-targeted systems and lowering anticancer drug toxicity.

## 1. Introduction

Biomedical research has been dedicating remarkable efforts to develop new therapeutic approaches providing for high selectivity and safety for the treatment of cancer, a disease that still represents the second cause of death worldwide, accounting for nearly 9.7 million deaths in 2022 (Source: WHO – accessed October, 2025).

Antibody‒drug conjugates (ADCs) have risen in prominence among the emerging therapeutic approaches for cancer treatment. Nevertheless, ADCs face different limitations, including poor tumor penetration, restricted efficacy in cancers with low receptor expression, and challenges in efficient patient screening, which can undermine clinical outcomes (Jain 1990; Thurber et al. 2008; Coats et al. 2019; Ocaña et al. 2020). Furthermore, the drug/antibody ratio (DAR) impacts the ADC features and performance. Indeed, a low DAR could reduce the antitumor efficacy, while high DAR may affect antibody structure, stability, and antigen binding, thereby causing a loss of activity (Tang et al. 2017).

To overcome the limitations of ADCs, targeted nanoparticle with drug conjugated on the surface, nanoparticle–drug conjugates (NDCs) that can accommodate substantially increased drug loading while offering tunable physicochemical properties have been investigated. Indeed, NDC can add up a high surface area for drug conjugation, and the peculiar physicochemical properties of the particle matrix can be selected to achieve specific responses to external stimuli (Farokhzad and Langer 2006; Peer et al. 2007; Hartshorn et al. 2018; Mitchell et al. 2021). However, while a high density of drugs on the NDC surface is advantageous, it implies a rational combination of stabilizing components.

Gold nanoparticles (GNPs) have recently progressed into clinical evaluation for tumor ablation and targeted delivery. Several colloidal gold nanomedicines have entered clinical trials (Zhang et al. 2023). In earlier work, we demonstrated that GNPs can also function as sonosensitizers, generating ROS upon ultrasound irradiation and inducing apoptosis (Brazzale et al. 2016).

Among the various targeting strategies used to endow drug nanocarriers with biorecognition and intracellular access, folate receptor (FR) targeting has been widely investigated because it is overexpressed on many solid tumor cells while remaining minimally expressed in most healthy tissues (Fernández et al. 2018). Recently, this approach has been clinically approved for drug delivery to ovarian cancer (Mirvetuximab soravtansine) (Moore et al. 2023).

Literature reports on GNP surface decoration with doxorubicin by electrostatic adsorption (Suarasan et al. 2016) or with doxorubicin conjugated to PEG via a hydrazone linker for controlled tumor release (Wang et al. 2011; Sun et al. 2014). Furthermore, systems with pH-releasable doxorubicin conjugated to GNPs through a short linker and shielded by a PEG coating have also been proposed (Aryal et al. 2009). These formulations suffer from non-specific cell uptake. The targeted doxorubicin-GNPs reported in the literature do not include folate as a targeting agent used in our work. Furthermore, these systems are frequently obtained by single-thiol anchoring of doxorubicin through short linkers, which results in lower stability with respect to the bidentate disulfide used in our study (Ruan et al. 2015). Targeted GNPs decorated with dendronized doxorubicin anchored through bidentate disulfide without PEG shielding did not yield sufficient cell selectivity (Dockery and Daniel 2023). Notably, none of the abovementioned studies reported the effects of drug loading on nanoparticle stability, release kinetics, and biological performance in 3D models. In previous works, we reported on folate-GNP ligand display and receptor-dependent cancer cell uptake (Mastrotto et al. 2011; Brazzale et al. 2016; Brazzale et al. 2017; Daniele et al. 2023). In these studies, we did not investigate the formulation constraints, such as doxorubicin loading capacity, colloidal stability or intracellular pH-triggered drug release, which are reported in the current study. Therefore, the novelty of our work lies in the development of upgraded GNPs obtained by integrating (i) folate targeting (through a PEG arm that allows for its exposure for folate receptor recognition) and (ii) a short lipoyl-hydrazone pH-cleavable linker anchored via a bidentate disulfide with increased conjugation stability of doxorubicin. Furthermore, a systematic formulation study was carried out to evaluate the relationship between the doxorubicin/GNP molar ratio and PEG coverage on the loading efficiency and colloidal stability. Finally, this study reports the biological performance of both targeted and untargeted formulations in 3D tumor spheroid models, providing the first direct comparative evidence of how folate targeting modulates infiltration and cytotoxicity in a physiologically relevant 3D environment. Notably, to the best of our knowledge, this is the first study that correlates the formulation composition with the colloidal and biopharmaceutical profiles of folated doxorubicin-loaded GNPs*.*

## 2. Materials and methods

### 2.1. Materials

Folic acid, sodium citrate tribasic dihydrate, tetrachloroauric(III) acid trihydrate, iodine, potassium iodide, barium chloride, N-hydroxysuccinimide (NHS), N,N′-dicyclohexylcarbodiimide (DCC), tris(2-carboxyethyl)phosphine hydrochloride (TCEP), triethylamine, α-lipoic acid, dimethylsulfoxide anhydrous (DMSO), chloroform, dichloromethane (DCM), diethyl ether (Et_2_O), Sephadex G-25 superfine, and Sephadex LH 20 gel filtration resins were purchased from Sigma-Aldrich (St. Louis, MO, USA). Analytical thin-layer chromatography (TLC) was carried out on glass sheets coated with silica gel (Merck F-254, Merck, Darmstadt, Germany). Doxorubicin hydrochloride (Doxo·HCl) was purchased from LC Laboratories (Woburn, MA, USA). BDP 630/650 X NHS ester was obtained from Lumiprobe (Hannover, Germany). NH_2_–PEG_2 kDa_–SH and NH_2_–PEG_3.5 kDa_–SH were purchased from JenKem Technology, USA (Plano, TX, USA). mPEG_2 kDa_–SH was acquired from Iris Biotech GmbH (Marktredwitz, Germany). ProLong Glass Antifade Mountant was purchased from Invitrogen Thermo Fisher Scientific (Waltham, MA, USA). All the chemical reagents used for cell culture, Dulbecco's modified Eagle's medium (DMEM), folic acid-free DMEM, RPMI-1640 medium, L-glutamine solution, D-(+)-glucose solution, sodium bicarbonate solution, fetal bovine serum (FBS), penicillin‒streptomycin solution, Trypan blue solution, and trypsin were supplied by Sigma-Aldrich (St. Louis, MO, USA). KB^FR+^ (HeLa derivative ECACC 94050408) and MCF7^FR−^ (human breast adenocarcinoma cell line) cells were obtained from the American Type Culture Collection (ATCC, Manassas, VA, USA).

### 2.2. Methods

Synthesis and characterization of the targeting agent FA–PEG_3.5 kDa_–SH (FA–PEG–SH), the fluorescent probe BDP–PEG_2 kDa_–SH (BDP–PEG–SH), and gold nanoparticles are reported in the Supporting Information.

#### 2.2.1. Synthesis of Doxo prodrug (proDoxo)

Step 1: α-lipoic acid (2.1 g, 10.0 mmol) was suspended in 50 mL of methanol and cooled in an ice bath under stirring for 15 min. Thionyl chloride (1.1 mL, 15.0 mmol) was added dropwise to the reaction mixture, which was stirred and refluxed at 75 °C for 18 h. After cooling to room temperature, the solvent was removed under reduced pressure, and the oily residue was dissolved in ethyl acetate. The solution was washed three times with a saturated sodium bicarbonate solution in a separator funnel. The organic phase was collected and dried over sodium sulfate. The organic solvent was removed under vacuum, and the oily residue was analyzed by ^1^H-NMR to assess the identity of the product. Yield: quantitative.

Step 2: Lipoyl methyl ester (2.2 g, 10.0 mmol) was dissolved under stirring in 65% hydrazine monohydrate (50 mL, 100.0 mmol), and the reaction mixture was heated under reflux for 1.5 h. After cooling, the reaction was quenched with a saturated ammonium chloride solution and extracted three times with chloroform. The organic phase was dried over sodium sulfate, and the solvent was evaporated under vacuum. The resulting yellow oil was purified by flash chromatography using a silica column eluted with a 90:10 v/v CHCl_3_/MeOH mixture containing 0.1 v/v% aqueous ammonia. Column fractions were spotted on silica TLC and stained (KMnO_4_) for visualization. The desired product was characterized by ^1^H-NMR. Yield: 98%.

Step 3: Lipoyl hydrazide (59 mg, 0.025 mmol) was dissolved in anhydrous methanol in a Schlenk tube. Doxo·HCl (58 mg, 0.01 mmol) and 100 µL of a 0.3% v/v mixture of TFA in anhydrous methanol were added to the reaction mixture. The mixture was stirred at room temperature in the dark for 3 days and monitored by RP-HPLC equipped with a Phenomenex Luna C18 column eluted in gradient mode with 10 mM ammonium acetate buffer, pH 6 (eluent A) and acetonitrile (eluent B). The amount of eluent B was increased linearly from 5 to 90% in 30 min, and the UV–vis detector was set at 488 nm. When the reaction achieved completion based on the disappearance of unreacted Doxo, the solvent was removed under vacuum. The crude product was purified by suspending in a 50:50 v/v methanol/diethyl ether mixture at approx. 1 mg/mL concentration, stirred and refluxed at 70 °C for 3 h and subsequently stirred at room temperature overnight. The conjugate was recovered as a solid material by filtration. The purity of the product was assessed by RP-HPLC. The final product was characterized by ^1^H-NMR spectroscopy and by LC‒MS mass spectroscopy by using an Acquity UPLC BEH C18 column, which was eluted with 10 mM ammonium acetate buffer, pH 6 (eluent A) and acetonitrile (eluent B), in gradient mode from 10% to 90% of eluent B in 15.5 min. The flow rate was set at 0.25 mL/min. The detection was performed in positive mode.

Lipoyl hydrazon Doxo (proDoxo): ^1^H NMR (300 MHz, DMSO-d_6_, δ, ppm): 1.17 (d, J = 7.0 Hz, 3 H, 6ʹ-CH_3_), 1.30−1.60 (m, 4H, 3"-CH_2_ and 4"-CH_2_), 1.63−1.78 (m, 2H, 2ʹ-Ha and 5"-Ha), 1.82−1.94 (m, 2H, 2ʹ-Hb and 5"-Hb), 2.00−2.18 (m, 2H, 8-CH_2_), 2.19−2.45 (m, 2 H, 2"-CH_2_), 2.70−2.78 (m, 2H, 7"-CH_2_), 2.89−3.04 (m, 2H, 10-CH_2_), 3.04−3.24 (m, 2H, 8"-CH_2_), 3.35−3.42 (m. 1H, 6"-H), 3.54−3.65 (m, 2 H, 3ʹ-H and 4ʹ-H), 4.00 (s, 3H, OCH_3_), 4.01−4.22 (m, 1H, 5ʹ-H), 4.38−4.46 (m, 1H, 14-Ha), 4.58 (d, J = 5.9 Hz, 14-Hb), 4.83−4.87 (m, 1H, OH), 4.96 (t, J = 4.7, 1H, 7-H), 5.30 (t, J = 5.9 Hz, 1H, 14-OH), 5.41−5.45 (m, 1H, 1ʹ-H), 5.51−5.55 (m, 1H, OH), 5.73−5.75 (m, 1H, NH), 7.67 (t, J = 8.0 Hz, 1H, 2-H), and 7.93 (d, H = 8.0 Hz, 2H, 1-H, 3-H).

LC-MS and ESI-TOF analysis: m/z lipoyl hydrazone Doxo [M-H^+^]: calculated = 746.24, found = 746.24.

#### 2.2.2. Functionalization of GNPs

The pH of 10 mL of 10 nM GNPs was adjusted to 9 by adding 0.1 M NaOH to avoid particle aggregation, as reported in the literature (Paciotti et al. 2004). Then, thiolated functional components were sequentially added as follows:

89.7 μL of a 56.9 μM FA-PEG-SH aqueous solution mixed with 19.3 μL of a 26.4 μM mPEG_2 kDa_–SH aqueous solution was added under stirring to the GNP samples to a final 50:5:1 [FA–PEG–SH/mPEG_2 kDa_–SH/GNP] molar ratio. After 3 h, 80.6 μL of a 25.3 μM BDP–PEG–SH aqueous solution was mixed with 30.9 μL of a 264 μM of mPEG_2 kDa_–SH aqueous solution and stirred to a final 20:80:1 [BDP–PEG–SH/mPEG_2 kDa_–SH]/GNP] molar ratio. After an additional 3 h, the GNP suspensions were added to increasing volumes of 2.85 mM lipoyl hydrazone Doxo (proDoxo) in mQ water to generate samples at increasing [proDoxo/GNP] molar ratio in the range 0–1500. Finally, after 3 h of incubation, the GNP surface was saturated by adding 40.6 μL of a 2.64 mM mPEG_2 kDa_–SH aqueous solution, yielding a 1000:1 [mPEG_2 kDa_–SH/GNP] molar ratio.

The conjugation yield of each functional component was derived by indirect measurements, and the non-conjugated fractions of each component were quantified. After each functionalization step, 1.5 mL of GNPs was added of 80 μL of saturated NaCl solution and frozen in liquid nitrogen to facilitate particle aggregation. Then, the particles were left to thaw at room temperature and centrifuged at 13,500 rcf for 30 min at 4 °C. 1.2 mL of supernatant was collected and freeze-dried. Then, the dried residue was redissolved in 400 μL of mQ water.

The supernatant collected after the incubation of the particles with FA-PEG-SH was spectrophotometrically analyzed to assess FA at 363 nm (Ɛ_363_ = 6,197 M^−1^ cm^−1^). The supernatant collected after the incubation with BDP–PEG–SH underwent spectrofluorometric analysis to assess BDP using ʎ_ex_ = 628 nm and ʎ_em_ = 642 nm. The BDP–PEG –SH concentration was derived from a calibration line. The supernatant collected after the incubation with proDoxo was spectrophotometrically analyzed at 488 nm to determine the Doxo concentration (Ɛ_488_ = 11,500 M^−1^ cm^−1^).

Finally, the supernatant collected after the addition of mPEG_2 kDa_–SH was analyzed by an iodine assay to quantify the unbound mPEG_2 kDa_–SH, referring to a calibration line.

The conjugation efficiency of each module was calculated as the difference between the fed moles of the functional agent and the unreacted moles detected in the supernatant.

#### 2.2.3. Size, size distribution, and zeta potential

The size of naked and functionalized GNPs was measured at 25 °C by dynamic light scattering (DLS) using a Malvern Zetasizer Ultra (Malvern Instruments Ltd., Malvern, UK) equipped with a red laser (10 mV, 633 nm) at a fixed angle of 173°. The data were analyzed using the ZS Xplorer software, version 3.22. Ten microliters of freshly prepared 10 nM GNPs underwent 100-fold dilution with 10 mM NaCl and 1 mM HEPES pH 7.4, and the sample was then analyzed. Size values were derived from an average of three DLS measurements in automatic mode. The results are expressed as the number distribution of the naked particles. The results are expressed as the intensity distribution for the functionalized particles.

Ζeta potential was assessed by Zetasizer Ultra analysis according to the provider's instructions. The 10 nM GNP suspension was analyzed after 100-fold dilution in 10 mM NaCl, 1 mM HEPS pH 7.4, using a disposable folder capillary (DTS1070) cuvette.

#### 2.2.4. TEM imaging

Transmission electron microscopic (TEM) images were obtained with a Tecnai G2 microscope FEI (FEI Tecnai, OR-USA). 2 nM naked or functionalized particles in mQ water were analyzed. The functionalized particles were negatively stained with 1% (w/v) uranyl acetate in Milli-Q water before analysis. The particle samples were placed on a carbon-coated copper grid and dried at room temperature. The average size of the particles was derived by measuring 200 individual particles with ImageJ software (Schneider et al. 2012).

#### 2.2.5. Release studies

##### 2.2.5.1. Hydrolysis of proDoxo.

A stock solution of proDoxo in DMSO at 20.6 mM was prepared. Dilutions (1 mL) at 200.6 μM in 10 mM ammonium acetate at pH 5 or 10 mM ammonium bicarbonate at pH 7.4 were generated. The samples were incubated at 37 °C during the study. Aliquots withdrawn at scheduled time points underwent mass analysis using a Micromass Q-TOF micro™ ESI-TOF mass spectrometry (Waters, Milford, MA, USA). The intensities of the proDoxo signal (746 m/z) and Doxo signal (544 m/z) were detected to determine the hydrolysis of proDoxo over time.

##### 2.2.5.2. Release of Doxo from GNPs.

One  milliliter of a 10 nM folate-targeted BDP-labeled gold nanoparticle dispersion functionalized with 1000 proDoxo molar ratios [FA–PEG–Doxo_1000_–GNPs] in 10 mM sodium phosphate at pH 7.4 or 10 mM ammonium acetate at pH 5 was loaded into a Float-A-Lyzer (MWCO 100 kDa). The dialysis membrane was transferred into a Falcon tube containing 20 mL of the same buffer supplemented with 0.5 w/V% Tween 80. At scheduled time points, the receiving buffer was removed, analyzed for fluorescence at *λ*_ex_ = 488 nm and *λ*_em_ = 590 nm and replaced with fresh buffer. Doxo concentration was derived from calibration lines obtained from the two release buffers in the 10–2000 nM concentration range.

#### 2.2.6. Cell culture

Human MCF-7^FR−^ breast adenocarcinoma (ICLC, Interlab Cell Line Collection, Genova, Italy) and human KB^FR+^ epidermoid carcinoma (ECACC, European Collection of Cell Culture, Salisbury, UK) cell lines were cultured as monolayer in RPMI 1640 and folate-free DMEM (FF-DMEM) growth medium, respectively, supplemented with 10% fetal bovine serum (V/V), 2 mM L-glutamine, 100.0 UI/mL penicillin and 100.0 μg/mL streptomycin in a humidified atmosphere containing 5% CO_2_ at 37 °C. The cells were detached using 0.05 w/V% trypsin-EDTA solution (Sigma Aldrich, St. Louis, MO, USA), resuspended in culture medium, and seeded at the appropriate cell densities for experiments.

#### 2.2.7. Cell association by flow cytometry

Folate-targeted BDP-labeled gold nanoparticles functionalized with increasing proDoxo molar ratios in the 0–1000 range [FA–PEG–Doxo–GNPs] or their non-targeted version [PEG–Doxo–GNPs] were tested to evaluate their association profile with KB^FR+^ cells.

KB^FR+^ cells were seeded in 24-well plates (1.5*10^5^ cells/well) and grown for 24 h in FF-DMEM supplemented with 10 V/V% FBS in a humidified 5% CO_2_ atmosphere. Afterward, the medium was removed, the cells were washed three times with PBS (w/o Ca^2+^/Mg^2+^) and then incubated with 2 nM of targeted FA–PEG–Doxo–GNPs or untargeted PEG–Doxo–GNPs in complete medium. After 2, 4, or 6 h of incubation, the particle-containing medium was removed, and the cells were washed three times with 200 μL/well of PBS. The cells were detached by treatment with 200 μL of 125 μg/mL of trypsin in PBS without Ca^2+^/Mg^2+^ and then collected in FACS tubes containing 200 μL of PBS, pH 7.4, 0.5 w/V% BSA, 5 mM EDTA, 2 mM NaN_3_, and 1% PFA for fixation. The cell samples were analyzed with a BD FACS Aria™ III flow cytometer (BD Biosciences, Franklin Lakes, NJ, USA) by setting the APC channel for BDP detection. 10^4^ events were recorded for each sample.

MCF-7^FR−^ cells were used as a negative control by virtue of their low FR expression (Gallon et al. 2015). MCF-7^FR−^ cells were seeded in 24-well plates (1.5*10^5^ cells/well) and grown for 24 h in FF-DMEM supplemented with 10% FBS in a humidified 5% CO_2_ atmosphere. Afterward, the medium was removed, the cells were washed three times with PBS (w/o Ca^2+^/Mg^2+^) and then incubated with 2 nM of targeted FA-PEG-Doxo-GNPs or untargeted PEG-Doxo-GNPs in the complete medium. After 6 h of incubation, the cells were washed three times with 200 μL of PBS. The cells were harvested as described previously and analyzed with a BD FACS Aria™ III flow cytometer by setting the APC channel for BDP detection. 10^4^ events were recorded for each sample.

#### 2.2.8. Cell uptake studies by confocal microscopy

KB ^FR+^ cells were seeded in a 24-well plate at 10^5^ cells/well density in FF-DMEM with 10% FBS on sterile glass slides and grown for 24 h under tissue culture conditions. The medium was removed, and the cells were incubated with 5 nM targeted FA–PEG–Doxo_1000_–GNP or untargeted PEG–Doxo_1000_–GNP dispersion or free Doxo at an equivalent drug concentration. The cells were treated with a pulse and chase scheme: the cells were incubated with particles or free drug for 2 or 4 h; the treatments were then removed, the cells were washed three times with PBS, and grown in fresh medium for 2, 4, or 24 h. The medium was removed, and the cells were washed three times with PBS without Ca^2+^/Mg^2+^ and fixed with 300 μL of 4 w/V% PFA for 15 min at room temperature. The cells were washed again with PBS without Ca^2+^/Mg^2+^. The nuclei were stained by treatment with 300 μL/well of DAPI (4.5 μg/mL in PBS) for 10 min at room temperature in the dark, after which the cells were washed three times with PBS. Afterwards, the glasses were mounted for confocal analysis by using 3 μL of mounting media. Confocal images were recorded using a Zeiss LSM800 confocal microscope and the ZEN software version 3.10 (Carl Zeiss Microscopy GmbH, Jena, Germany). The fluorophores were visualized using the 355 nm laser for DAPI detection (nuclei), a 488 nm laser for Doxo, and a 633 nm laser for BDP detection.

#### 2.2.9. Spheroids of KB^FR+^ cells

Multicellular spheroids of KB^FR+^ cells were generated using the liquid overlay method (Gaio et al. 2016). Briefly, KB^FR+^ cells were harvested from monolayer cultures by trypsinization and seeded in flat-bottomed 96-well plates (650 cells/well) previously coated with 1 w/V% agarose in DMEM to prevent cell adhesion. Immediately after seeding, the plates were centrifuged at 200 rcf for 5 min to promote cell aggregation and then transferred to an incubator under the cell culture conditions described above. After 3 days, the spheroids reached a diameter of about 400 μm and were used for the following experiments. The spheroids were incubated with 100 μL/well of fresh FF-DMEM containing 10% FBS and increasing concentration of targeted FA–PEG–Doxo_1000_–GNP or untargeted PEG–Doxo_1000_–GNPs or free Doxo in the 0.5–5 µM range of equivalent drug concentration. After 6 h of incubation, the spheroids were washed three times with PBS and maintained in FF-DMEM with 10% FBS in the incubator for an additional 66 h. Cell viability was measured using the CellTiter-Glo® 3D Cell Viability Assay. Briefly, 50 μL of cell medium was left in each spheroid well, and 50 μL of CellTiter-Glo® 3D Reagent was added; the well content was mixed by mild shaking for 5 min, incubated at room temperature for 25 min, and luminescence was measured with a Perkin Elmer Envision instrument (Waltham, MA, USA).

Moreover, for selected conditions, the spheroid morphological changes induced by the treatments were monitored before and after the 72-h treatment using a bright field microscope (DMI6000B, Leica, Wetzlar, Germany). The software LAS AF Lite (Leica Microsystems) was used to analyze spheroid images and derive the minimum diameter (*d*_min_) and maximum diameter (*d*_max_) and calculate the spheroid volume using Equation (1):

(1) $$V=\frac{\pi *d_{min}*d_{max}}{6}.$$

The spheroid volume ratio (R) was then calculated using Equation (2):

(2) $$R=\frac{V_{i}}{V_{0}}*100,$$

where *V*_i_ is the spheroid volume measured after the 6-h contact with treatments and 66 h of additional growth in medium, and *V*_0_ is the spheroid volume before the treatment.

Spheroids treated with 5 nM of FA–PEG–Doxo_1000_–GNPs or PEG–Doxo_1000_–GNPs underwent confocal fluorescence imaging with CLSM after 6, 18, and 24 h of continuous treatment. Doxo and BDP were detected using the lasers at 488 nm and 633 nm, respectively.

#### 2.2.10. Statistical analysis

The results are presented as the mean ± SD (*n* = 3). The ANOVA test was used for the statistical analysis of significance, followed by a Tukey's HSD (honestly significant difference) test as a post hoc test. (GraphPad Prism 9): * indicates *p* < 0.05, ** indicates *p* < 0.01, *** indicates *p* < 0.001, and **** indicates *p* < 0.0001, ns indicates no statistically significant differences.

## 3. Results and discussion

The tumor-targeting and controlled release of doxorubicin were obtained by combining five functional components: i. Gold nanoparticles (GNPs) as a colloidal platform for surface decoration, ii. Folate–PEG_3.5 kDa_–SH as a targeting agent, iii. the fluorescent tag BODIPY X–PEG_2 kDa_–SH, for trackability iv. the pH-responsive prodrug of doxorubicin lipoyl–hydrazone–doxorubicin, v. mPEG_2 kDa_–SH for colloidal stability and stealth features.

GNPs with an average diameter of 15 nm have been used because of their high surface/volume ratio and easy surface conjugation of thiol-terminating components through strong metal‒sulfur bonds (Bard et al. 2018). Furthermore, studies reported in the literature have shown that GNPs with size ≤20 nm coated with hydrophilic polymers exhibit prolonged blood half-life and EPR effect (Dong et al. 2019).

Folic acid was selected, among other targeting agents, because it is a small, non-immunogenic molecule with well-defined chemistry, high affinity for its receptor (KD ~ 10⁻¹⁰ M) (Sudimack and Lee 2000), and excellent stability, which make it particularly suitable for reproducible and scalable nanoparticle functionalization. Furthermore, its overexpression in many cancer cells compared to healthy cells and endocytic mechanism make it an excellent candidate to yield selective tumor targeting and drug delivery (Leamon and Low 1991). Alternative ligands such as antibodies, peptides, and aptamers are highly tumor-selective but generally suffer from high production costs, batch-to-batch variability, and reduced stability during nanoparticle processing and may interfere with nanoparticle size or surface packing constraints, an essential advantage for achieving deep tumor penetration of nanoparticles.

Folated GNPs are taken up by folate receptor-overexpressing cancer cells through receptor-mediated endocytosis (Paulos et al. 2004; Kim et al. 2017; Daniele et al. 2023). The Doxo pH-sensitive prodrug (ProDoxo) was obtained by drug conjugation to lipoic acid through a hydrazone bond. The 1,2 thiolane function of lipoic acid allows for anchoring of the prodrug on the GNP surface while the hydrazone bond allows for drug-controlled release in the acidic environment of intracellular organelles, namely, endolysosomes (Wahbeh and Milkowski 2019; Yang et al. 2020; Cheng et al. 2023).

The 3.5 kDa PEG was selected based on previous evidence that combining it with stealthing 2 kDa PEG enables effective folate–receptor biorecognition in cancer cells (Daniele et al. 2023), and its backbone length of ~80 oxyethylene units was expected to provide sufficient ligand exposure on the GNP surface to support this interaction. The 2 kDa PEG was selected for endowing stealth properties and RES avoidance characteristics for nanoparticles for parenteral administration. 2 kDa PEG-coated nanocarriers have been clinically approved for drug delivery (Beltrán-Gracia et al. 2019; Roces et al. 2020; Padín-González et al. 2022).

### 3.1. Folate–PEG_3.5 kDa_–SH, BDP–PEG_2 kDa_–SH and lipoyl–hydrazone*–*doxorubicin synthesis

Folate–PEG_3.5 kDa_–SH (FA) was synthesized according to a procedure developed by our group (Brazzale et al. 2016; Daniele et al. 2023). The chemical identity of Folate–PEG_3.5 kDa_–SH was confirmed by mass spectrometry analysis, which showed the characteristic bell-shaped profile of the conjugate cantered at 4,117 m/z, while purity determined by RP-HPLC analysis was >98%.

The fluorescent probe derivative used for GNP labeling was obtained by BDP conjugation to the amino end group of a thiol-terminating 2 kDa PEG (Scheme S1). The shorter polymer length compared to that used for folic acid conjugation was chosen to avoid BDP exposure on the GNP surface in order to avoid interference with folate receptor binding while ensuring that the BDP fluorescence remains uncompromised by GNP quenching (Brazzale et al. 2016; Daniele et al. 2023). The identity of the conjugate was confirmed by ^1^H NMR and mass spectrometry. The derived integral ratio between the NMR characteristic proton signals of BDP and PEG resulted in a 0.9:1 BDP/PEG molar ratio conjugation yield (Figure S1)**.** The mass spectrum was cantered at 2,448 m/z (Figure S2), and the purity determined by RP-HPLC analysis was >99%.

**Scheme 1.**
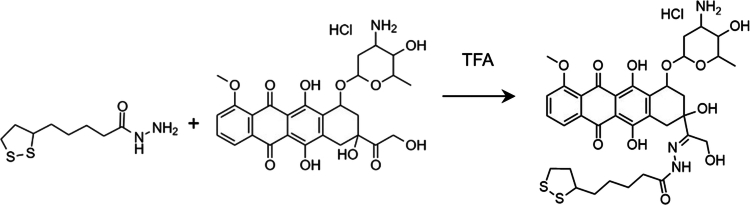
Synthesis of the proDoxo by conjugation of Doxo to lipoyl hydrazide.

The releasable Doxo prodrug lipoyl‐hydrazone*‐*doxorubicin was obtained by drug conjugation to the hetero bifunctional linker lipoyl-hydrazide according to a three-step procedure: 1. conversion of lipoic acid to lipoyl methyl ester (Scheme S2); 2. conversion of the methoxy group of lipoyl methyl ester to the hydrazide derivative (Scheme S3); and 3. conjugation of the lipoyl hydrazide (in slight excess) to the ketone of Doxo in the presence of TFA as a reaction catalyst (Scheme 1). The chemical identities of the intermediates lipoyl methyl ester and lipoyl hydrazide were derived as detailed in the Supporting Information (Figures S3–S5). The reaction conditions used in the process guaranteed both high yield (>70% for proDoxo over three steps) and high purity (>98% by NMR) of the final isolated compound.

**Figure 1.**
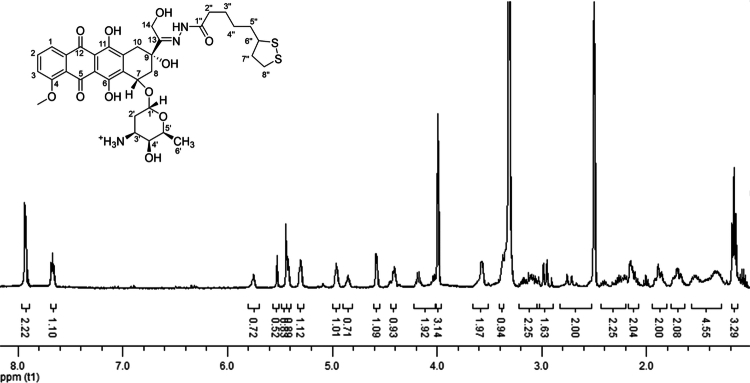
^1^H-NMR spectra of proDoxo recorded in DMSO-d_6_. See the experimental section for peak assignments.

The Doxo conjugation to the linker monitored by RP-HPLC was complete in 72 h. The reaction mixture was easily purified by digestion in diethyl ether, resulting in a proDoxo isolated yield of 82%. The ESI-TOF mass spectroscopy signal at m/z 746.24 matched the theoretical [M–H^+^] molecular weight of the prodrug (Figure S6), and the RP-HPLC analysis showed 98.7% purity. The identity of the proDoxo was confirmed by ^1^H-NMR (Figure 1).

### 3.2. Synthesis, coating, and characterization of decorated GNPs

Gold nanoparticles (GNPs) with a 15.2 ± 1.3 nm mean diameter and 0.15 ± 0.05 PDI (Figure S8A) were produced under controlled pH by reduction of HAuCl_4_ according to a revised method from the literature (Li et al. 2011). The negative zeta potential (−39.9 ± 3.3 mV) is due to the citrate used for HAuCl_4_ reduction.

The GNPs were surface-functionalized according to a multistep procedure involving sequential nanoparticle incubation with the following steps: 1. FA–PEG–SH, 2. BDP–PEG–SH, 3. proDoxo, and 4. mPEG–SH. The UV–vis spectra of the GNPs after each functionalization step show that conjugation does not alter the absorption profile of the GNPs, which retain a consistent surface plasmon resonance at 520 nm, indicating the absence of particle aggregation (Figure S7).

The thiol group of FA-PEG-SH efficiently displace loosely bound citrate molecules on the GNP surface, resulting in stable surface-targeting agent conjugation (Ulman 1996; Kumar et al. 2012; Perera et al. 2018): 50:1 FA–PEG–SH/GNP molar ratio yielded over 98% FA–PEG–SH conjugation. This degree of FA decoration was previously shown to provide optimal cancer cell recognition and GNP uptake (Brazzale et al. 2017; Daniele et al. 2023). Similarly, the 20:1 BDP–PEG–SH/GNP molar ratio yielded over 99% BDP–PEG–SH conjugation, which provided effective fluorescent labeling of GNPs without excessive surface coverage [BDP-labeled folate-targeted GNP (FA-GNP)].

**Figure 2.**
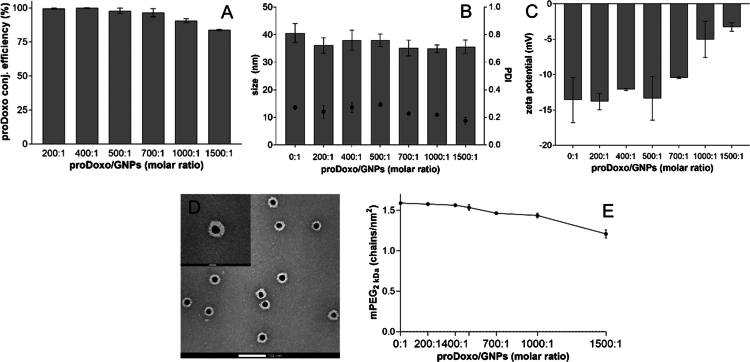
Characterization of the GNPs. (A) Conjugation efficiency of proDoxo at increasing proDoxo/FA-GNP molar ratios. (B) Size distribution (bars) of FA-PEG-Doxo-GNPs functionalized with increasing proDoxo/FA-GNP molar ratios. The black dots (●) represent the PDI and refer to the right y-axis. (C) Zeta potential of FA–PEG–Doxo–GNPs functionalized with increasing proDoxo/FA-GNP molar ratios. (D) TEM images of functionalized FA–PEG–Doxo–GNPs obtained with 1000:1 proDoxo/FA-GNP molar ratio. Scale bars in the figure: 100 nm; scale bar in the top left insert: 20 nm. (E) PEG density on FA–PEG–Doxo–GNPs obtained at increasing proDoxo/GNP molar ratios.

Doxo was conjugated to BDP-labeled FA-GNPs by using the proDoxo prodrug, which contains a hydrolysable hydrazone bond that allows for drug release, and a terminating lipoyl group, which allows for GNP surface attachment. In order to identify the product with the highest drug loading, colloidal stability, and cell-targeting efficiency, a library of BDP-labeled conjugates [FA–Doxo–GNP] was generated by using proDoxo/FA-GNP molar ratios in the range of 0–1500:1. The prodrug conjugation efficiency was nearly quantitative in the 0:1–700:1 proDoxo/FA-GNP molar ratio range feed and decreased to 84% for the 1500:1 proDoxo/FA-GNP feed molar ratio, probably because of the steric hindrance from the bound molecules (Figure 2A).

FA-Doxo-GNPs were stabilized by surface conjugation of mPEG_2 kDa_–SH using 1000:1 mPEG_2 kDa_–SH/FA–Doxo–GNP molar ratio. The proDoxo/GNP molar ratio did not significantly affect the FA–PEG–Doxo–GNP library member size (Figure 2B), which was in the range of 35–40 nm, and the PDI was below 0.3 for all formulations. However, the negative charge of the nanoparticles slightly decreased as the proDoxo/GNP molar ratio increased (Figure 2C) because of citrate displacement by proDoxo conjugation. Furthermore, the Doxo amino group (pKa of 9.93) (Alves et al. 2017) is predominantly (~80%) protonated at pH 7.4 (the test condition), thus contributing to a decrease in the negative charge on the nanoparticle surface.

Transmission electron microscopy (TEM) images showed the spherical shape of FA–PEG–Doxo–GNPs and the polymer coating on their surface (Figures 2D, S8 and S9).

PEGylation of the GNPs was aimed at obtaining nanosystems with stealth properties. The *mushroom* or *brush* PEG conformation on nanoparticle surfaces depends on the PEG chain length and density. As the density increases, steric hindrance also increases, leading to a decrease in opsonization (Schroffenegger et al. 2020; Storjohann et al. 2023). The conjugation efficiency of mPEG_2 kDa_–SH to FA–Doxo–GNPs was affected by steric hindrance from proDoxo molecules on the GNP surface. As the proDoxo/FA-GNP ratio increased from 200:1 to 1500:1, the PEG_2 kDa_ conjugation efficiency decreased from 95% to 70% (Table S1 and Figure 2E). Since saturation was achieved at an mPEG_2 kDa_–SH/GNP molar ratio of 1000:1, we chose this latter ratio for the production of particles to be investigated in further studies. Based on PEG chain density on the FA–PEG–Doxo–GNP surface, we calculated that the distance among chains (D) is between 0.792 and 0.909 nm, while the Flory radius (R_F_) of PEG_2 kDa_ reported in the literature is 2.8 nm (Ma et al. 2014). Since all the GNP library formulations have D < R_F_, a dense brush conformation is expected, which explains the homogeneous size of the nanoparticles. When PEG is organized in the *brush* conformation, it forms a corona on the particle surface, which was confirmed by the TEM images obtained with all formulation compositions (Figures 2D, S8 and S9). Additionally, the dense *brush* conformation of the PEG_2 kDa_ coating may prevent the folate from collapsing on the particle surface, thus favoring the targeting agent exposure on FA-PEG-Doxo-GNP for cell biorecognition.

The effect of bound Doxo on FA–PEG–Doxo–GNP stability in the presence of serum proteins was investigated. Indeed, hydrophobic molecules on the nanoparticle surface (e.g. Doxo; LogP 1.41 (Radeva et al. 2023)) can favor aggregation, which can affect the safety and biopharmaceutical properties of the nanoparticles, including targeting. A proDoxo/FA-GNP molar ratio of up to 1000:1 yielded stable FA-PEG-Doxo-GNPs in serum for 72 h at 37 °C, while higher feed ratios caused an increase in nanoparticle size in the presence of serum proteins (Figure S10). Additionally, the results reported in Figure 2A show that above 1000:1 proDoxo/FA-GNP molar feed ratio, the drug loading efficiency significantly decreases. Therefore, the 1000:1 Doxo/GNP formulation was selected to yield the highest drug loading and colloidal stability of the folate-targeted Doxo-loaded GNPs*.*

### 3.3. Drug release

**Figure 3.**
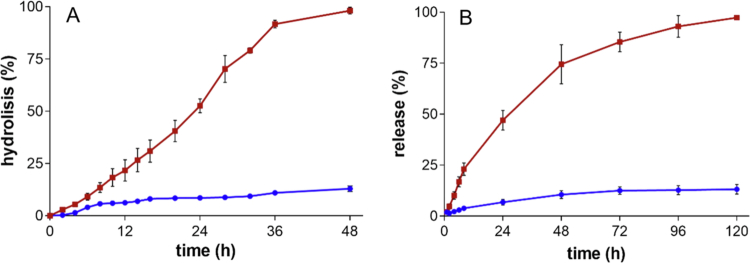
pH-dependent release of Doxo. (A) Hydrolytic stability profile of proDoxo in aqueous solution performed by mass spectrometry. The samples were incubated at pH 5 (light brown) or 7.4 (blue) at 37 °C. (B) Doxo release profile from FA–PEG–Doxo_1000_–GNPs evaluated by dialysis and quantified by spectrofluorimetry. The samples were incubated at pH 5 (light brown) or 7.4 (blue) at 37 °C.

The Doxo release due to hydrazone bond cleavage was investigated at pH 7.4 and 5.0, which mimic blood and endosomal pH conditions, respectively. At pH 5.0, 100% Doxo was released from the prodrug proDox in 48 h (Figure 3A), while in the same timeframe, only 13% of Doxo was released at pH 7.4.

The Doxo release from FA–PEG–Doxo–GNPs formulated with 1000:1 proDoxo/FA-GNP molar ratio showed the same pH-dependent behavior as that of proDoxo. At pH 5.0, 74% of the drug was released within 48 h, and complete drug release was achieved within 120 h (Figure 3B). A few studies reported in the literature show that GNPs with sizes similar to those of FA–PEG–Doxo–GNPs undergo lysosomal trafficking, where their accumulation persists for more than 2 weeks (Balfourier et al. 2020), which is compatible with the five-day drug release from FA–PEG–Doxo–GNPs detected at conditions mimicking the pH of endolysosomes.

The slower release of GNP-conjugated Doxo compared to the hydrolysis of the free prodrug (proDoxo, Figure 3A) at pH 5 reflects the complexity of the release process, which involves multiple sequential steps, including hydrazone bond cleavage, desorption of Doxo from the nanoparticle surface, and diffusion through the polymeric corona. This complexity seems to have a lower impact at pH 7.4, probably because of the slow drug release from both proDoxo and FA–PEG–Doxo–GNPs.

The data reported in Figure 3B show that the FA–PEG–Doxo–GNPs undergo limited drug release in the blood at pH 7.4, reaching a plateau of about 10% of the loaded drug in 72 h. Notably, pharmacokinetic data reported in the literature show that in mice, PEG–GNPs of ~38 nm in diameter exhibit a plasma half-life of ~14.6 h (Cai et al. 2007). Over this time, the amount of drug released from FA–PEG–Doxo–GNPs was less than 5% of the amount released from the conjugated doxorubicin (Figure 3B).

The hydrazone linker behaved as expected, showing limited drug release at pH 7.4 and cleavage at pH 5. Comparable or even slower hydrazone-based release kinetics have been reported for other Doxo-GNP systems, which nonetheless retain strong activity in cellular and in vivo tumor models (Zhou et al. 2011; Tao et al. 2021). Moreover, many slow- to intermediate-growing tumors may benefit from sustained intracellular drug availability. The confirmed slow release of Doxo from GNPs in blood pH-mimicking conditions (pH 7.4) is crucial for preventing systemic toxicity upon parenteral administration. However, other complex investigations should be performed to elucidate other determinants of in-blood fate.

Although in biological environments various mechanisms can cooperatively contribute to drug release, in blood and endolysosomal compartments, where the GNPs were found to localize (Brazzale et al. 2017), thiol–exchange release is negligible owing to very low glutathione concentrations (1–6 μM) (Pisoni et al. 1990; Franceschini et al. 2014; Hakuna et al. 2015). Furthermore, the lipoyl bidentate anchoring further stabilizes prodrug attachment to GNP surface compared to monothiol anchoring derivatives. Overall, these considerations support the relevance of our pH-triggered release model.

### 3.4. Cellular association by flow cytometry

**Figure 4.**
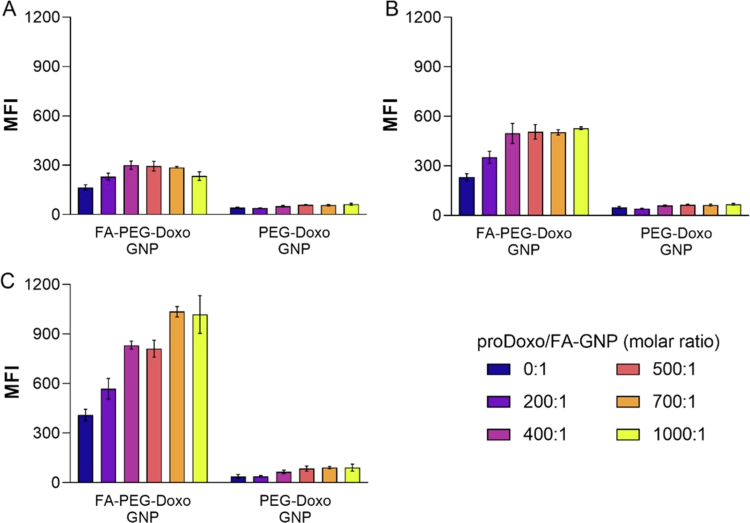
Cellular association profiles of KB^FR+^ cells incubated with BDP-labeled targeted FA-PEG-Doxo-GNPs and untargeted PEG–Doxo–GNPs for (A) 2, (B) 4, or (C) 6 h. Targeted or untargeted GNPs were decorated with increasing molar ratios of proDoxo, as reported in the legend. The results are reported as the means of three independent experiments ± standard deviations. MFI values refer to BDP fluorescence.

The targeting ability of decorated BDP-labeled FA–PEG–Doxo–GNPs was investigated by flow cytometry using KB^FR+^ human cervical carcinoma cells that overexpress the folic acid receptor (FR) (Rathinaraj et al. 2015; Siwowska et al. 2017). PEG–Doxo–GNPs (FA-free nanoparticles) were used as a negative control. The median BDP fluorescence intensity (MFI) of KB^FR+^ was assessed at three timepoints (2, 4, and 6 h) (Figure 4). Cytofluorometric analysis demonstrated that FA–PEG–Doxo–GNPs selectively associated with KB^FR+^ cells at significantly higher extent compared to PEG–Doxo–GNP. FA–PEG–Doxo–GNP association with KB^FR+^ cells was found to occur in a time-dependent profile. After 6 h of cell incubation, FA–PEG–Doxo–GNPs and PEG–Doxo–GNPs yielded at least 88% and less than 8.4% positive cell counts, respectively (Figure S11). Furthermore, an increasing trend in cell association was observed along the particle library as the proDoxo density on the nanoparticle surface increased. This trend plateaued for the particles with the highest proDoxo densities at all the tested time points either for folated or nonfolated nanoparticles (Figures 4 and S12). This behavior can be attributed to the decrease in absolute negative zeta potential with increasing proDoxo density on the nanoparticle surface, which reduces electrostatic repulsion with negatively charged KB^FR+^ cell membranes (Fuchigami et al. 2021). However, this nonspecific effect plays a limited overall role in cell interaction and does not compromise the selectivity conveyed by the targeting agent.

The targeted particles obtained with a 1000:1 proDoxo/FA-GNP molar ratio [FA–PEG–Doxo_1000_–GNP] were selected among the library formulation members as the best compromise between the cellular association efficiency and Doxo loading. After 6 h of incubation with cells, this formulation displayed an 11.1-fold higher association with KB cells than the untargeted PEG–Doxo_1000_–GNP counterpart.

**Figure 5.**
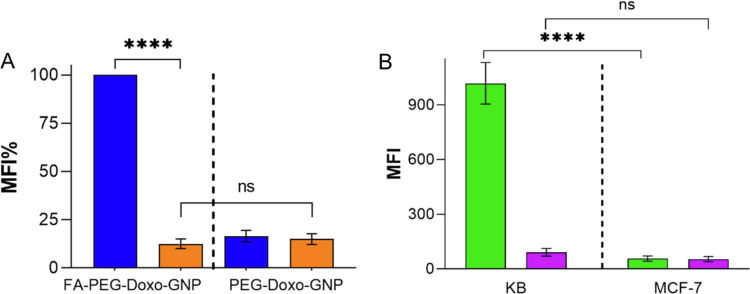
(A) Competition association profile of KB^FR+^ cells incubated with targeted FA–PEG–Doxo_1000_–GNPs or non-targeted PEG–Doxo_1000_–GNPs for 6 h in folic-free medium (blue) or in medium containing 1 mM folic acid (orange). The results are normalized to the targeted GNPs incubated with KB^FR+^ cells in folic-free medium, which represents 100% cellular association. The results are reported as the means of three independent experiments ± standard deviations. MFI values refer to BDP fluorescence (****p < 0.0001). (B) Cellular association profile of targeted FA–PEG–Doxo_1000_–GNPs (green) or non-targeted PEG–Doxo_1000_–GNPs (purple) formulations with KB^FR+^ and MCF-7^FR−^ cells. Results are reported as means of three independent experiments ± standard deviations. The MFI values refer to BDP fluorescence. (****p < 0.0001).

The FR-driven cell association of targeted nanoparticles was confirmed by competitive studies. Co-incubation of FR overexpressing KB^FR+^ cells with FA–PEG–Doxo_1000_–GNPs and 1 mM folic acid strongly reduced the cell up-take of the nanoparticles while in the case of untargeted PEG–Doxo_1000_–GNPs the nanoparticle up-take was not affected by the presence of folic acid (Figure 5A).

Finally, similar cell association profiles were obtained by incubating low FR expressing human breast cancer cell line MCF-7^FR-^ (Gallon et al. 2015) with targeted FA-PEG-Doxo_1000_-GNPs and non-targeted PEG-Doxo_1000_-GNPs, which further confirmed that the folated nanoparticles were selectively taken up by the cells by FR targeting (Figure 5B).

### 3.5 Cell uptake by confocal microscopy

FR-targeted nanocarriers are internalized and trafficked into the endolysosomes (Kamen and Caston 1986), where the acidic microenvironment can provide drug release through cleavage of pH sensitive bonds.

**Figure 6.**
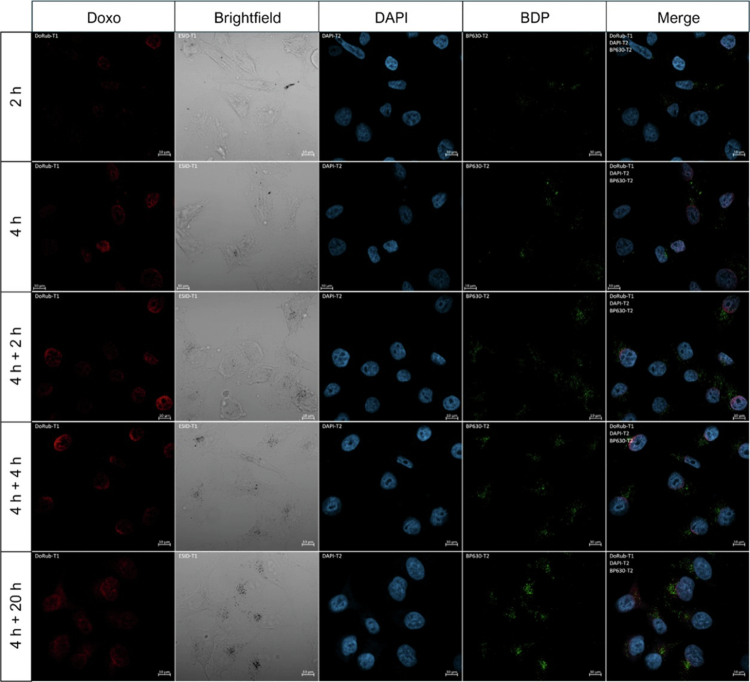
Confocal laser microscopic images of KB^FR+^ cells incubated with BDP-labeled FA–PEG–Doxo_1000_–GNPs for 2 or 4 h (first and second rows, respectively). The equivalent concentration of Doxo was 5  μM. The cells were incubated for 4 h, after which they were washed, followed by further 2 or 4 or 20 h of growth in fresh medium (third to fifth rows, respectively). The cells were stained with DAPI (light blue) for nuclei. Particles are detected by BDP fluorescence, which is displayed in green, while Doxo fluorescence is displayed in red. A 40× objective was used in the microscope for imaging. Scale bars on panels: 10 µm.

The KB^FR+^ cell uptake and trafficking of FA-PEG-Doxo_1000_-GNPs was investigated by confocal microscopy. Confocal images related to BDP fluorescence showed time-dependent nanoparticle internalization; intracellular FA-PEG-Doxo_1000_-GNPs were detected after 2 h of incubation, and the nanoparticle uptake increased over the time, which was consistent with the cytofluorimetric data (Figure 6; first and second rows). In contrast, the fluorescence related to Doxo was barely detectable in the cells after 2 h of FA-PEG-Doxo_1000_-GNP incubation, indicating slow drug release. Doxo associated with the nuclei was detected after 4 h of cell incubation with FA-PEG-Doxo_1000_-GNPs, while KB^FR+^ cell incubation with free Doxo resulted in remarkable drug disposition in the cell nuclei after 2 h (Figure S13).

The sustained intracellular release of Doxo was investigated by pulse and chase treatment (Figure 6, third to fifth rows). After 4 h of FA–PEG–Doxo_1000_–GNP incubation with KB^FR+^ and subsequent nanoparticle removal, the BDP green fluorescence in the cell cytosol remained unchanged over the 20 h of observation, as expected. Persistent Doxo fluorescence was also visible in the nuclei at different timepoints, indicating the sustained release of Doxo. In contrast, the treatment of KB^FR+^ cells with an equimolar concentration of free Doxo did not result in prolonged drug association with the cell nuclei (Figure S13).

**Figure 7.**
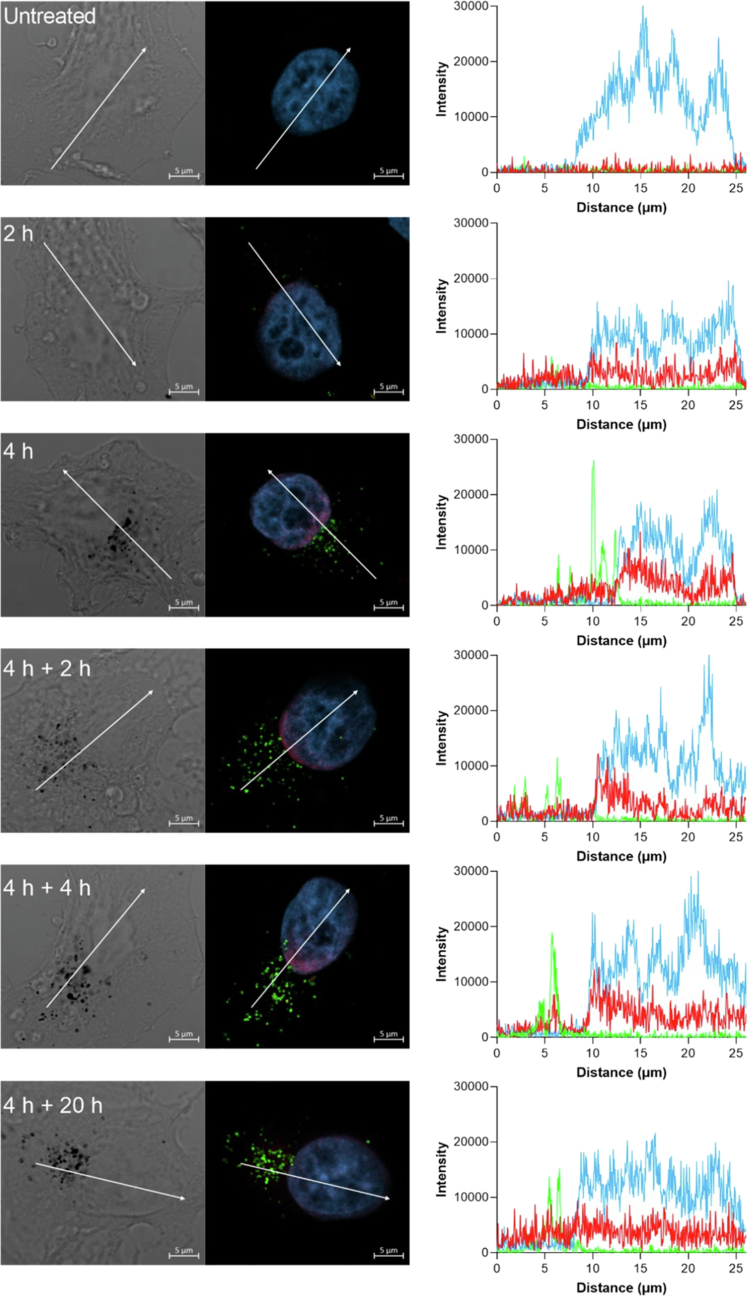
Line scanning analysis of confocal microscopic images of KB^FR+^ cells incubated with BDP-labeled FA–PEG–Doxo_1000_–GNPs at equivalent concentration of Doxo (5 μM). The cells were incubated for 4 h, after which they were washed, followed by further 2 or 4 or 20 h of growth in fresh medium. The images in the first column are bright field images; the images in the second column are confocal fluorescence images. The cells were stained with DAPI (light blue) for nuclei; the particles were detected by the BDP fluorescence displayed in green; and the Doxo fluorescence is displayed in red. A 40× objective was used in the microscope for imaging. Scale bars on the panels: 5 µm. The figures on the third column present line scanning analysis of the corresponding cell on the left: blue line for DAPI; green line for the particles; and red line for Doxo.

Notably, the line scanning analysis performed on the confocal images of KB^FR+^ cells confirmed that after 2 h of incubation, the red fluorescence of Doxo released from FA–PEG–Doxo_1000_–GNPs was already detected in the nuclei and increased over the incubation time (Figure 7). After 4 h of pulsed incubation with FA–PEG–Doxo_1000_–GNPs and along the 4 h chase, a gradient of Doxo-related fluorescence along the nucleus derived from the Doxo released by FA–PEG–Doxo_1000_–GNPs in proximity of the nucleus was observed. After 20 h of GNP removal, the red fluorescence was more homogeneously diffused throughout the cell, including the cytosol, as a result of sustained drug release from the FA–PEG–Doxo_1000_–GNPs. Notably, the bright field images show dark spots corresponding to the metallic particles that fairly correlate with the green fluorescence spots of BDP-labeled particles.

### 3.6. Cytotoxicity study

**Figure 8.**
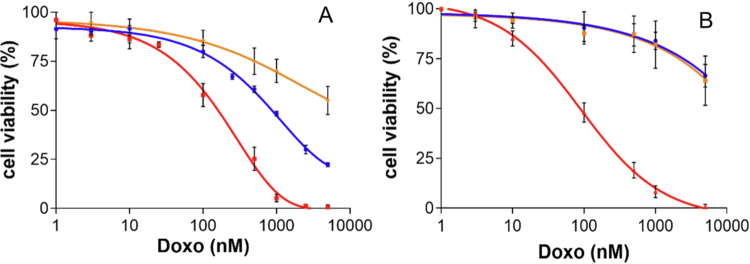
Viability profiles of KB^FR+^ (A) and MCF-7^FR−^ (B) cells treated with FA–PEG–Doxo_1000_–GNPs (blue), or PEG–Doxo_1000_–GNPs (orange), or free Doxo (red). The cell viability was derived by MTT assay after 6 h of incubation with the treatments and 66 h after removal of the treatments. The tested equivalent Doxo concentrations ranged from 1 nM to 5 μM.

Cell viability studies were performed using MCF-7^FR−^ and KB^FR+^ (Figure 8). In both cell lines, free Doxo was more cytotoxic than the targeted and nontargeted nanoparticles.

In MCF-7^FR−^ cells, Doxo had an IC_50_ 99.8 nM, while both FA–PEG–Doxo_1000_–GNPs and PEG–Doxo_1000_–GNPs showed similar dose-dependent cytotoxic profile, with IC_50_ > 5000 nM. The cell viability profiles obtained with FA–PEG–Doxo_1000_–GNPs and PEG–Doxo_1000_–GNPs are consistent with the low FR expression in MCF-7^FR−^ cells. In the case of KB^FR+^ cells, Doxo had an IC_50_ of 117.3 nM, while FA–PEG–Doxo_1000_–GNPs had an IC_50_ of 671.6 nM. The non-targeted PEG–Doxo_1000_–GNPs showed remarkable lower cytotoxicity compared to the targeted nanoparticles, with a cytotoxicity profile and IC_50_ similar to those obtained with MCF-7^FR−^ cells.

These results are in agreement with the previous discussion, consolidating the active targeting of FA–PEG–Doxo_1000_–GNPs and the pH-dependent drug release that takes place in the endosomal compartment upon nanoparticle internalization. The 5.7-fold lower IC_50_ of targeted FA–PEG–Doxo_1000_–GNPs compared to free Doxo obtained in KB^FR+^ cells may be attributed to the slow drug release, which provides a lower availability of the cytotoxic agent than the free drug. However, the sustained drug release from FA–PEG–Doxo_1000_–GNPs upon cell internalization provides for cell exposure to the drug for a longer time after treatment, resulting in enhanced therapeutic efficacy.

### 3.7. Cancer spheroid treatment with GNPs

The disposition of BDP labeled FA–PEG–Doxo_1000_–GNPs and the Doxo delivery into cancer tissues was investigated by using spheroids of KB^FR+^ cells, which closely simulate the three-dimensional architecture and environment of tumors in vivo (Bromma et al. 2021). Although FA–PEG–Doxo_1000_–GNPs showed selective cell uptake, the distribution into the tumor tissue may be hindered by the access of the nanoparticles in the tumor matrix (Ni et al. 2015; Solomon et al. 2016; Bugno et al. 2016; Millard et al. 2017). Therefore, FA–PEG–Doxo_1000_–GNPs were designed with a suitable size for infiltration into tumors. Studies reported in the literature have shown that nanoparticle penetration is inversely correlated with particle size, with particles around or smaller than 50 nm possessing better infiltration abilities in tumor tissues and being homogeneously distributed (Tang et al. 2013; Tang et al. 2014).

**Figure 9.**
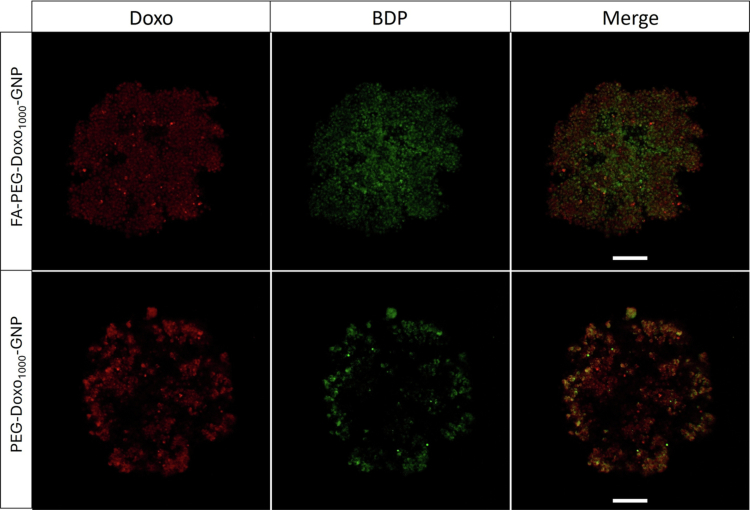
Confocal microscopic images of spheroids of KB^FR+^ cells after 18 h of incubation with BDP-labeled targeted FA–PEG–Doxo_1000_–GNPs (upper panel) or untargeted PEG–Doxo_1000_–GNPs (lower panel). Images represent the fluorescence of the median stack of the spheroids. Particles were detected by BDP fluorescence and are displayed in green, and Doxo fluorescence is displayed in red. Scale bars in the panels: 200 μm.

The results obtained by KB^FR+^ spheroid incubation with FA–PEG–Doxo_1000_–GNPs showed more uniformly distributed BDP-related fluorescence with respect to the untargeted PEG–Doxo_1000_–GNPs, indicating a more homogeneous disposition of the former in the spheroid matrix (Figure 9). In contrast, untargeted GNP appear more associated with the outer rim of the spheroid. Furthermore, the Doxo-related fluorescence detected in spheroids treated with FA–PEG–Doxo_1000_–GNPs also showed a homogeneous distribution within the spheroid. In contrast, the untargeted PEG–Doxo_1000_–GNPs yielded inhomogeneous Doxo-related fluorescence, which was consistent with the distribution profile in spheroids. Dedicated and in-depth studies are needed to elucidate the reasons for the diverse dispositions of folate-targeted and nontargeted Doxo-coated GNPs and the possible implication of cellular viability, receptor-related mechanisms and intercellular permeability over time.

**Figure 10.**
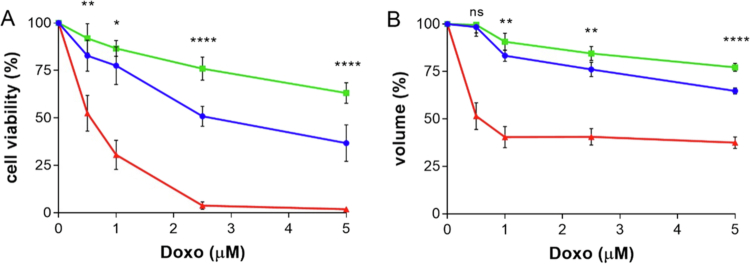
Biological studies on spheroids of KB^FR+^ cells. (A) Cell viability of spheroids of KB^FR+^ cells treated with targeted FA–PEG–Doxo_1000_–GNPs and untargeted PEG–Doxo_1000_–GNPs. Cell viability was assessed by the CellTiter-Glo 3D assay. (B) KB cell spheroid volume reduction after treatment. The spheroids were incubated for 6 h with FA–PEG–Doxo_1000_–GNPs (blue), PEG–Doxo_1000_–GNPs (green), or free drug (red) at increasing equimolar concentrations of Doxo in the 0.5–5 µM range. Spheroid viability and volume were measured after removal of the treatments and 66 h of growth in fresh medium. Statistical analysis refers to targeted *vs.* untargeted nanoparticles: *p* < 0.0001 (****), 0.0002 (***), 0.0021 (**), 0.0332 (*), and 0.1234 (ns).

The different cytotoxicities obtained with free Doxo, targeted, and untargeted nanoparticles correlate with the reduction in spheroid volume (Figure 10B). Indeed, FA–PEG–Doxo_1000_–GNPs resulted in a 26% reduction in the volume of the spheroid, which corresponds to a 1.6 times higher therapeutic efficacy compared to the untargeted PEG-Doxo_1000_-GNPs. The different profiles of the FA–PEG–Doxo_1000_–GNPs with respect to the untargeted GNP result from a combination of more homogeneous disposition within the spheroid observed by confocal microscopy, thus allowing for homogeneous biorecognition of the folate receptor, and higher cell association and uptake with more efficient drug release upon exposure to the endolysosome acid environment.

Notably, the therapeutic performance of drug delivery systems for active tumor targeting depends on several factors, including the bloodstream circulation profile, stability, passive disposition in the tumor tissue, diffusion in the extracellular matrix, and degree of expression and homogeneity of the cell target. Folate receptor-α (FRα) expression varies substantially across solid tumors, both among different tumor types and within individual lesions. Quantitative and immunohistochemical studies have demonstrated strong FRα overexpression in most epithelial ovarian and selected endometrial carcinomas, whereas lung adenocarcinoma, triple-negative breast cancer, pancreatic cancer, and colorectal carcinoma display more variable and often heterogeneous expression patterns (Parker et al. 2005; Kelemen 2006; Hartmann et al. 2007; Shia et al. 2008; Zhang et al. 2014; Agodi et al. 2015; Ledermann et al. 2015; Gonzalez et al. 2024). Even in FRα-high-expressing tumors, focal or patchy intratumoral staining is frequently reported (Parker et al. 2005; Shia et al. 2008; Ledermann et al. 2015). This biological heterogeneity has direct implications for folate-targeted nanocarriers because the cellular uptake of folate-decorated constructs depends on receptor density (Wang et al. 2023). Therefore, accurate personalized FRα expression assessment (e.g. immunohistochemistry or FR-targeted imaging) should be carried out to select the optimal drug delivery system (Moore et al. 2023).

## 4. Conclusions

FA–PEG–Doxo–GNPs have been demonstrated to be effective NDC for the targeted and controlled delivery of Doxo to cancer cells. The large surface area of gold nanoparticles was exploited to conjugate targeting agents, drugs at the highest density, and stabilizing polymers. The accurate design of the composition, including the GNP core size, allows for obtaining the nanosystem with suitable biopharmaceutical features, such as colloidal stability, drug loading, targeting, safety, and GNP clearance from the bloodstream.

Compared to existing liposomal Doxo formulations, the system we developed offer higher performance. Doxyl® is highly stable under physiological conditions and releases only ~15%–18% of its payload over 120 h at pH 7.4, with no relevant pH-dependent effects (Russell et al. 2018). Thus, it does not offer pH-triggered release within endolysosomes (Seynhaeve et al. 2013). In contrast, FA–PEG–Doxo–GNPs support controlled intracellular delivery. With respect to penetration, Doxyl typically accumulates at the tumor rim with limited diffusion due to its large size (Biswas et al. 2013; Tchoryk et al. 2019); in contrast, the colloidal and targeting features of FA–PEG–Doxo–GNPs provide a more homogeneous distribution in tumor spheroids with respect to the non-targeted counterparts. This resulted in more efficient therapeutic performance with respect to non-targeted nanoparticles, as demonstrated by the reduction of the spheroid volume and cancer cell killing.

Although this work establishes the formulation principles and identifies the main parameters governing the biopharmaceutical properties and in vitro performance of FA–PEG–Doxo-GNPs, clinical translation may face challenges related to the complexity of individual physiopathological conditions, including the tumor type, developmental stage, growth rate, heterogeneity of target expression, and other patient-specific factors. Therefore, future steps toward translation will require a structured preclinical program including comprehensive safety assessment, pharmacokinetics, biodistribution, and comparative efficacy studies in relevant animal models to evaluate the applicability of these formulations across different cancer models.

In early work, we demonstrated that gold nanoparticles act as efficient sensitizers for the sonodynamic treatment of cancer (Brazzale et al. 2016). The promising results obtained in the present study provide new perspectives for combining site-selective anticancer chemotherapy with localized ultrasound-based physical treatment (Foglietta et al. 2025). This integrated approach offers a compelling opportunity to achieve enhanced and more selective therapeutic effects through controlled drug release and the spatial confinement of the physical stimulus.

## Supplementary Material

Supplementary material Supporting_Drug Del_Revision_no track

## Data Availability

The data that support the findings of this research are available from the corresponding author, SS, upon reasonable request.
