# Supplementary material for: Folate-targeted gold nanoparticles for doxorubicin delivery in tumor spheroids
Source: Drug Deliv. 2025 Dec 30;33(1):2607390. doi: 10.1080/10717544.2025.2607390 (PMC12777817; doi:10.1080/10717544.2025.2607390)
Supplement: Supplementary material — Supporting_Drug Del_Revision_no track [file IDRD_A_2607390_SM0950.docx]

Supporting Information

**Folate-targeted gold nanoparticles for Doxorubicin delivery in tumor spheroids**

Raffaella Daniele, Agnese Fragassi, Cristiano Pesce, Marco Verona,

Giovanni Marzaro, Stefano Salmaso*, Paolo Caliceti

**1. Methods**

- 1. **Synthesis of the Folate-PEG_3.5kDa_-SH**

The targeting agent used in this project, namely Folate-PEG_3.5kDa_-SH (FA-PEG-SH), was synthesized according to a procedure previously reported with some modifications [1,2]. In brief, folic acid (50 mg, 0.113 mmol) was dissolved in 1 mL of anhydrous DMSO. N-hydroxysuccinimide (NHS, 19 mg, 0.169 mmol) was added to the solution, followed by dicyclohexylcarbodiimide (DCC, 35 mg, 0.169 mmol). The reaction mixture was kept under gaseous nitrogen and stirred for 18 h at room temperature in the dark. Then, the crude product was filtered to remove insoluble dicyclohexylurea using a sintered glass funnel. After filtration, the activated N-hydroxy succinimidyl-folate ester was isolated by precipitation in diethyl ether and extensively washed to remove DMSO. N-hydroxy succinimidyl-folate ester (25 mg, 0.046 mmol) and NH_2_-PEG_3.5kDa_-SH (54.1 mg, 0.015 mmol) were dissolved in 1 mL of anhydrous DMSO containing triethylamine (2.10 μL, 0.015 mmol). The reaction mixture was stirred for 12 h at room temperature in the dark. Then, the crude product was isolated by precipitation in diethyl ether and purified by size exclusion chromatography using Sephadex G-25 superfine resin eluted with 1 mM ammonia solution (pH 9) as the mobile phase. Eluted fractions were tested for PEG and folate presence by spotting the fractions on thin-layer chromatography plates; folate was detected by a UV-Vis lamp and PEG by exposing the plates to Iodine vapor in a closed chamber. Fractions positive to both tests were pooled and lyophilized to obtain a fine yellow powder.

The product was then exposed to reducing conditions to regenerate the thiol-bearing conjugate from the dimerized derivative. To this aim, the crude product (20 mg, 8.2 μmol) was dissolved in 5 mL of 50 mM ammonium acetate buffer at pH 5, followed by Tris(2-carboxyethyl)phosphine (TCEP, 20.5 mg, 82 μmol) and stirred for 3 h. Then, the reaction mixture was transferred into a Spectra/Por® Float-a-lyzer® G2 (MWCO 0.5-1 kDa) and dialyzed against 1 mM HCl/1 mM EDTA for 2 days in the dark. The final product FA-PEG-SH was lyophilized and characterized by UV-Vis spectroscopy at 363 nm (molar extinction coefficient of folate = 6,197 M^-1^cm^-1^) to assess folate [3,4] and by Iodine assay to quantify the PEG [5]. The conjugation yield was calculated as the ratio between the folic acid and PEG and was 98 % mol/mol. The purity of the conjugate was assessed by RP-HPLC using a Phenomenex Luna C18 column eluted in gradient mode of Milli-Q water (eluent A) and acetonitrile added of 0.05% TFA (eluent B). The eluent B was increased linearly from 5 to 90 % over 35 minutes, and the UV-Vis detector was set at 363 nm. The purity was confirmed to be > 98 %.

The chemical identity of the product was confirmed by MALDI mass spectroscopy by using sinapinic acid as the matrix (m/z [M-H^+^]= 4,117), and by ^1^H NMR (300 MHz, DMSO-d_6_, δ, ppm): δ 8.64 (s, 1H, CH of FA), 7.64 (m, 2H, aromatic of FA), 6.65 (m, 2H, aromatic of FA), 4.35 (m, 1H, CH(COOH) of FA), 3.50 (s, CH_2_O of PEG, ∼180H), 2.89 (t, 2H, J = 6.4Hz, CH_2_-S).

- 1. **Synthesis of BDP-PEG_2kDa_-SH (BDP-PEG)**

BDP-X-NHS (10 mg, 15.5 µmol) was dissolved in 500 µL of anhydrous DMSO and sequentially added of NH_2_-PEG_2kDa_-SH (25.7 mg, 12.8 µmol) and triethylamine (1.8 µL, 12.8 µmol). The reaction was maintained under stirring for 18 h at room temperature in the dark. Afterward, the crude product was purified by size exclusion chromatography using Sephadex LH20 resin and absolute ethanol as the mobile phase. Column fractions were spotted on thin-layer chromatography plates and BDP and PEG presence was visualized by UV lamp irradiation and plate exposure to iodine vapor in a closed chamber, respectively. Fractions positive to both tests were pooled in a round bottom flask, and the solvent was removed under reduced pressure. The purified product (20.7 mg, 8.45 µmol) was dissolved in 5 mL of 50 mM ammonium acetate at pH 5 containing Tris(2-carboxyethyl)phosphine (TCEP, 21.1 mg, 84.5 μmol) and stirred for 3 h in the dark. The mixture underwent lyophilization, and the solid BDP-PEG_2kDa_-SH (BDP-PEG-SH) was characterized by UV-Vis spectroscopy at 628 nm (molar extinction coefficient of BDP-X = 97,000 M^-1^cm^-1^, based on provider data: Lumiprobe, Hannover - Germany) to assess BDP concentration, and by Iodine assay to determine PEG concentration. The conjugation yield, expressed as [BDP]/[PEG] molar ratio, was estimated to be 96%. The recovery yield was 88.5 mol%.

The purity of the conjugate was assessed by RP-HPLC using a Jasco HPLC system equipped with a reverse phase column Phenomenex Luna C18 eluted in a gradient mode with mQ water containing 0.05% of TFA (eluent A) and acetonitrile containing 0.05% of TFA (eluent B). Eluent B was increased linearly from 10 to 90 % in 15 min, and the fluorescent detector was set at ʎ_ex_=628 nm and ʎ_em_=642 nm.

The chemical identity of the product was confirmed by MALDI spectrometry (m/z [M-H^+^] = 2,448) and by ^1^H NMR spectroscopy: (400 MHz, DMSO-d_6_, δ, ppm): δ 2.88 (2H, t, J = 5.1 Hz), 2.19 (2H, t, J = 7.4 Hz), 7.61 (1H, dd, J = 6.3, 1.2 Hz), 6.87 (1H, d, J = 7.5 Hz), 6.78 (1H, dd, J = 1.1, 0.5 Hz), 7.17 (2H, t, J = 8.8, 1.9, 0.5).

- 1. **Synthesis of gold nanoparticles**

Gold nanoparticles (GNP) were generated using the Turkevich method with modifications [6]. All the glassware was washed with aqua regia (3:1 V/V of 12.2 M HCl / 14.6 M HNO_3_) and then rinsed with Milli-Q water before use. 200 μL of 1 mM HAuCl_4_ solution in Milli-Q water and 3.08 mL of 20 mM sodium hydroxide were added to 16.72 mL of Milli-Q water. The mixture was heated to 85 °C under stirring. Then, 240 μL of 1.7 M trisodium citrate solution in Milli-Q water was added to the reaction mixture and stirred for 30 min. The GNP suspension was left to cool down at room temperature and extensively characterised in terms of size, polydispersity, and concentration.

The concentration of GNP suspension was assessed according to the procedure reported by the literature [7-9] and our previous studies [1]. First, the molar extinction coefficient (ε_506_), referred to the absorbance of the particle samples at 506 nm, was derived using **Equation S1**:

${ln\varepsilon}_{506}=k*lnD+a$ **Equation S1**

where *D* is the diameter of the nanoparticles measured by DLS (size distribution by number), and *a* and *k* are two constants whose values are 10.80505 and 3.32111, respectively.

Then, the molar concentration of the GNP was calculated according to the Lambert-Beer equation **(Equation 2)**:

$GNPs (M)=\frac{A_{506}}{\varepsilon_{506}*b}$ **Equation S2**

where A_506_ is the absorbance of the sample at 506 nm, ε_506_ is calculated by Equation S1, and *b* is the path length of the quartz cuvette.

- 1. **PEG density on GNP surface**

The conjugation efficiency of mPEG_2kDa_-SH to GNP was used to derive the number of PEG chains associated to the GNP surface. The PEG density was calculated based on a 15 nm diameter particle with a surface area approximated to the one of a sphere and was calculated as the ratio between the number of PEG chains anchored to GNP and the surface area. The resulting values were used to derive the distance (D) between each grafting point using **Equation S3**:

$D=\sqrt{1/\sigma}$ **Equation S3**

where σ is the PEG density on the GNP surface (chains/nm^2^).

- 1. **Colloidal stability**

The particles’ stability was evaluated in FF-DMEM supplemented of 10% FBS by size analysis over time using a Malvern Zetasizer Ultra. Targeted PEG-Doxo-(FA-BDP)-GNP decorated with increasing proDoxo/(FA-BDP)-GNP in the 200-1500:1 molar ratio range were analyzed.

2 mL of 10 nM PEG-Doxo-(FA-BDP)-GNP was centrifuged at 13,500 rcf for 30 minutes at 4 °C. 1.8 mL of supernatant was removed, and 200 μL of mQ water was added, resulting in 50 nM GNP. Then, GNP were transferred in 2-mL low-binding Eppendorf tubes and diluted to 2 nM with folic-free DMEM supplemented of 10% FBS. The particles were maintained at 37 °C in the dark and analyzed by DLS at scheduled time points. Immediately before the size analysis, the GNP underwent 100-fold dilution with mQ water. The DLS analysis was run using the fluorescence filter of the Zetasizer Ultra to remove any fluorescence interference. The intensity-weighted mean value and polydispersity index were recorded as the average of three measurements.

- 1. **Cell viability assay**

KB^FR+^ cells were seeded in a 96-well plate in folic free-DMEM supplemented with 10% FBS at a density of 1*10^4^ cells per well (200 μL/well) and allowed to adhere and grow for 24 hours under tissue culture conditions. Then, the medium was replaced with 200 μL of PEG-Doxo_1000_-(FA-BDP)-GNP or PEG-Doxo-(BDP)-GNP or Doxo in complete medium at increasing equivalent Doxo concentrations in the 0.001-5 µM range. Cells were incubated for 6 hours at 37 °C in 5 % CO_2_ atmosphere. Afterwards, the treatments were removed, cells were washed twice with 200 μL of PBS w/o Ca^2+^/Mg^2+^ and medium was replaced with 200 μL/well of fresh FF-DMEM containing 10% FBS and cells were grown for a further 66 hours. Then, the medium was removed, and cells were washed twice with PBS, and 200 μL/well of medium with 20 μL of MTT solution (5 mg/mL in PBS without Ca^2+^/Mg^2+^) was added in each well. Plates were incubated for 3 hours at 37 °C. The medium was removed, and 200 μL of DMSO was added to each well to dissolve the formazan crystals generated by the live cells. The plates were gently shaken for 15 min, and the spectrophotometric absorbance was measured at 570 nm using a INNO-M microplate spectrophotometer (Ltek, Republic of Korea). The cell viability was derived using untreated cells as a reference.

Cytotoxicity of targeted PEG-Doxo-(FA-BDP)-GNP, non-targeted PEG-Doxo-(BDP)-GNP and free Doxo was investigated on the control MCF-7^FR-^ cell line using the same protocol used for KB ^FR+^ cells.

1. **Results**

**Table S1.** Conjugation efficiency of the mPEG_2kDa_-SH to GNP coated with increasing proDoxo/(FA-BDP)-GNP feed molar ratios.

| proDoxo/(FA-BDP)-GNP molar ratio | mPEG_2kDa_ conjugation efficiency (%) | mPEG_2kDa_ density (chains/nm^2^) | mPEG_2kDa_ chain distance D (nm) |
| --- | --- | --- | --- |
| 0:1 | 98.3 | 1.59 | 0.792 |
| 200:1 | 96.3 | 1.58 | 0.796 |
| 400:1 | 95.1 | 1.56 | 0.800 |
| 500:1 | 93.2 | 1.54 | 0.807 |
| 700:1 | 88.9 | 1.46 | 0.826 |
| 1000:1 | 86.4 | 1.44 | 0.834 |
| 1500:1 | 70.4 | 1.21 | 0.909 |

- 1. **Synthesis and characterization of BDP-PEG-SH**

BDP-PEG_2kDa_-SH was obtained by conjugating N-hydroxysuccinimidyl ester-activated Bodipy X (BDP-NHS) to NH_2_-PEG_2kDa_-SH **(Scheme S1)**.


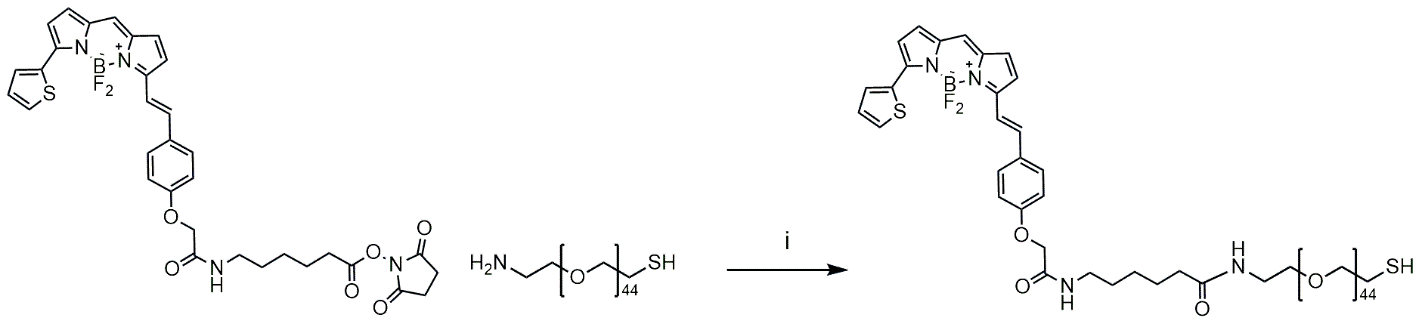


**Scheme S1**. Synthesis of BDP-PEG. (i) anhydrous DMSO, 1 eq TEA.

BDP-NHS conjugation to NH_2_-PEG_2kDa_-SH was performed using a molar excess of the activated fluorophore to promote the conjugation to the NH_2_-PEG_2kDa_-SH amino group. ^1^H NMR spectrum of BDP-PEG is reported in **Figure S1.** MALDI-TOF spectra of NH_2_-PEG_2kDa_-SH and BDP-PEG are reported in **Figure S2**.


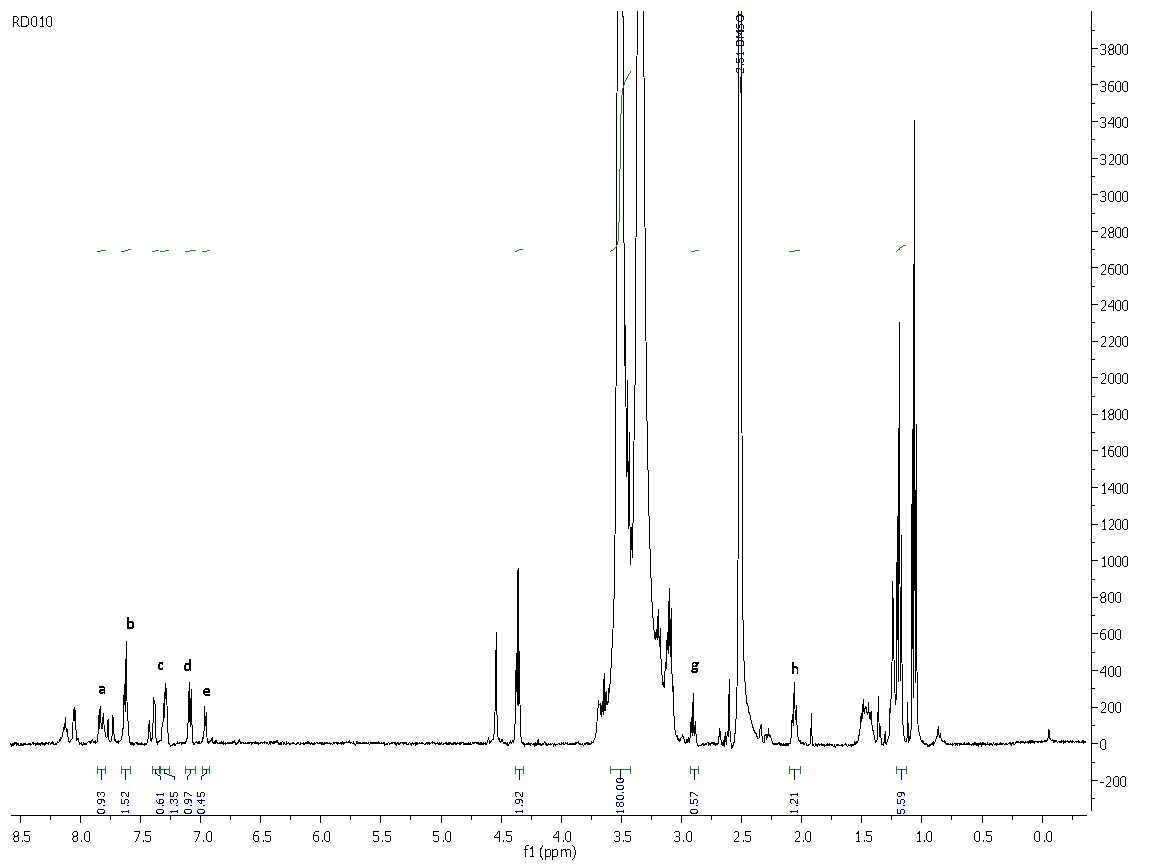

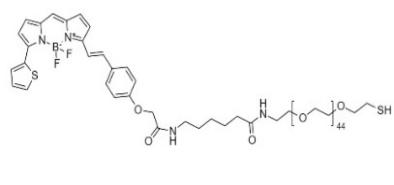

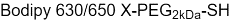


**a**

**b**

**c**

**e**

**c**

**c**

**c**

**h**

**f**

**g**

**d**

**f**

**Figure S1**. ^1^H NMR spectrum of BDP-PEG_2kDa_-SH in DMSO-d_6_ with signal assignment.


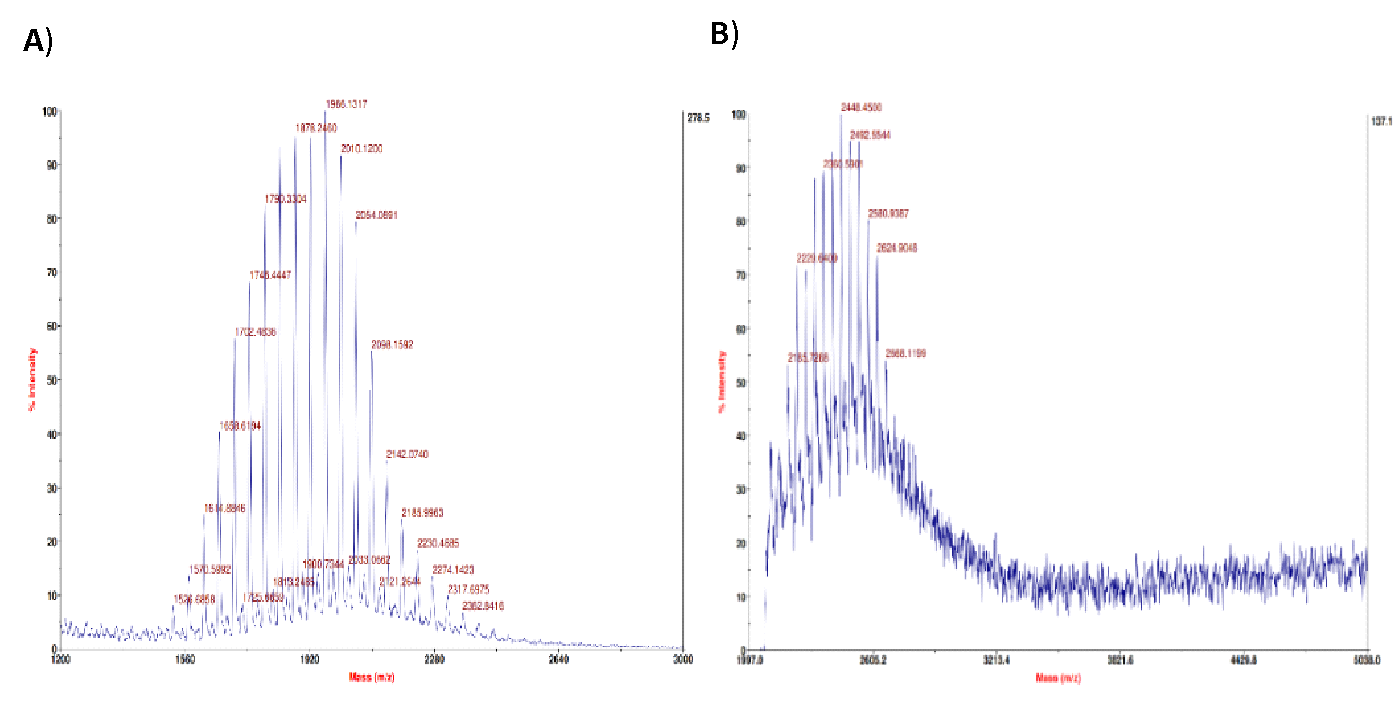


**Figure S2**. MALDI-TOF spectra of A) NH_2_-PEG_2kDa_-SH (m/z for [M-H^+^] = 1,966) and B) BDP-PEG_2kDa_-SH conjugate (m/z for [M-H+] = 2,448).

- 1. **Synthesis of the lipoyl methyl ester**

Lipoic acid was activated using thionyl chloride. The reaction was carried out at 0 °C to avoid degradation induced by the exothermic reaction. The acyl chloride intermediate reacts with MeOH, resulting in the formation of methyl ester (**Scheme S2**).


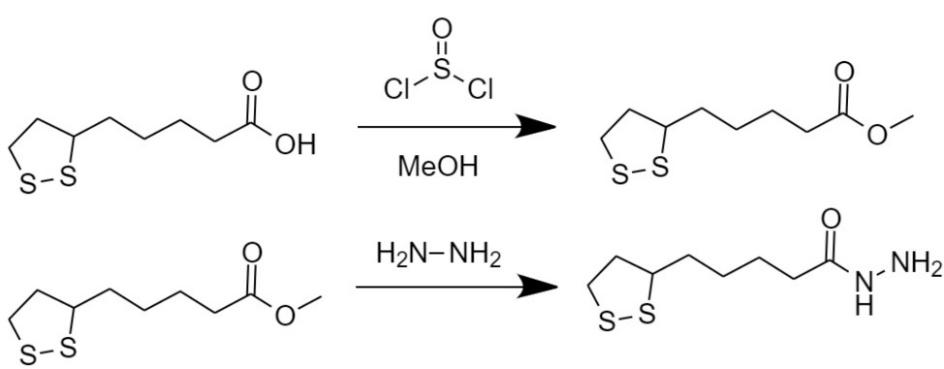


**Scheme S2**. Synthesis of the lipoyl methyl ester.

A yellow oil was obtained in quantitative yield. The esterification of lipoic acid to the corresponding methyl ester was confirmed by ^1^H-NMR spectroscopy.  The esterification resulted to be complete, as demonstrated by the disappearance of the carboxylic group signal at 12.01 ppm of the lipoic acid (**Figure S3**) and the formation of a new signal of the methyl ester at 3.58 ppm, overlapped with the “a” proton signal at 3.60 ppm (**Figure S4**). Moreover, the signal of the two protons “g” shifted to higher chemical shifts, from 2.21 ppm for the lipoic acid to 2.28 ppm after the reaction, because these protons are α-protons adjacent to the carbonyl group involved in the esterification process.


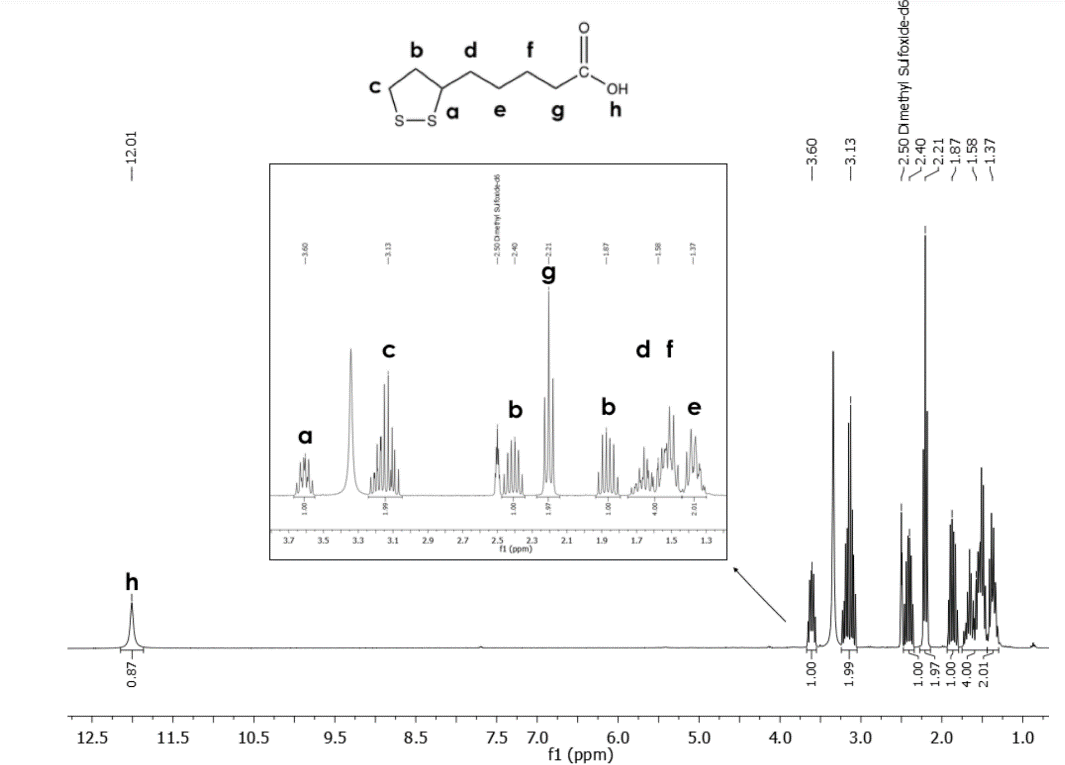


**Figure S3.** ^1^H NMR spectrum of α-Lipoic acid performed in DMSO-d_6_ with signal assignment.

*
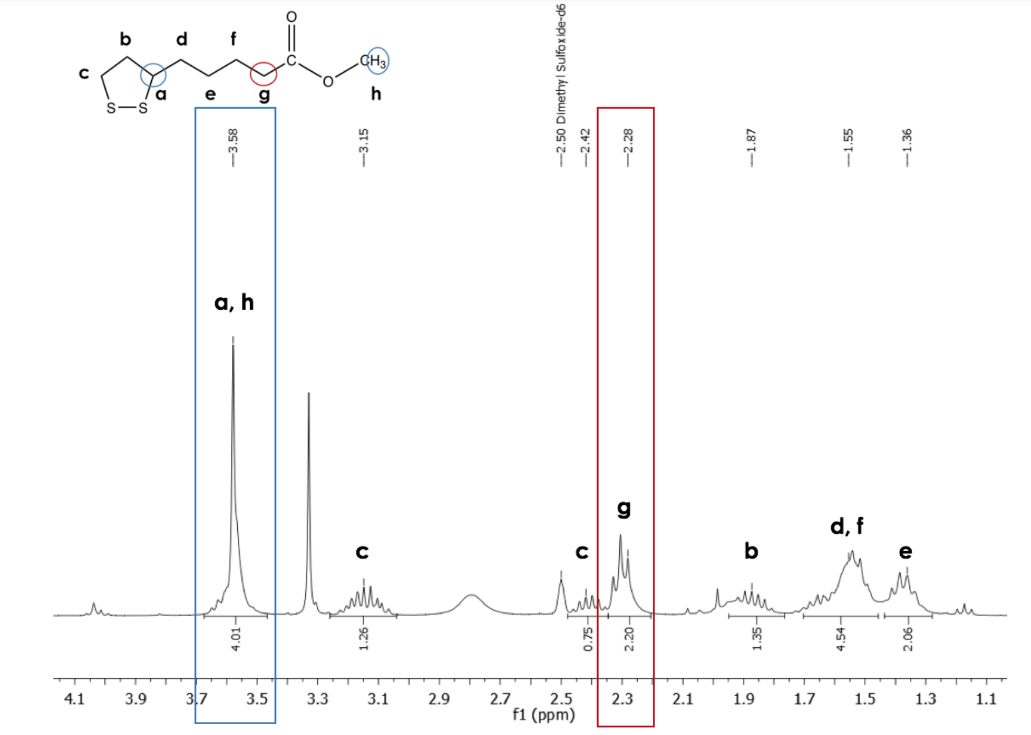
*

**Figure S4.** ^1^H NMR spectrum of Lipoyl methyl ester performed in DMSO-d_6_ with peak assignment.

**1.4 Synthesis of the lipoyl hydrazide**

Lipoyl methyl ester was converted into lipoyl hydrazide by refluxing with hydrazine monohydrate (**Scheme S3**).


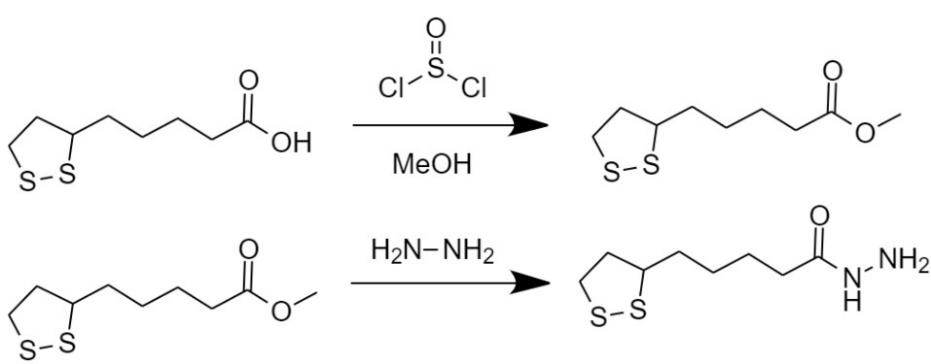


**Scheme S3**. Synthesis of the lipoyl hydrazide.

After 1.5 hour of reaction, the yellow oil of the crude product was purified by a chromatographic column (98% yield). ^1^H-NMR analysis was performed (**Figure S5**) to investigate the chemical identity and conversion yield. ^1^H-NMR spectrum shows the disappearance of the ester signal at 3.58 ppm (only the signal of proton “a” at 3.60 ppm remained) and the shift of the protons “g” signal from 2.28 ppm of the ester derivative to 2.00 ppm in the hydrazide one.


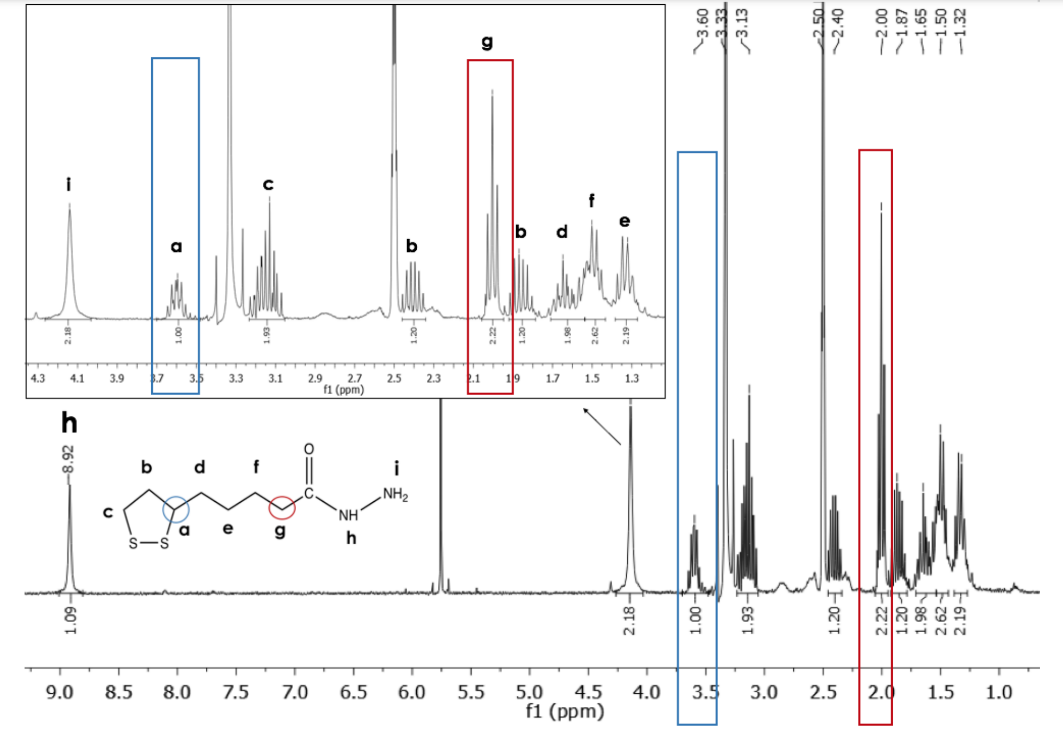


**Figure S5**. ^1^H NMR spectrum of Lipoyl hydrazide performed in DMSO-d_6_ with peak assignment.


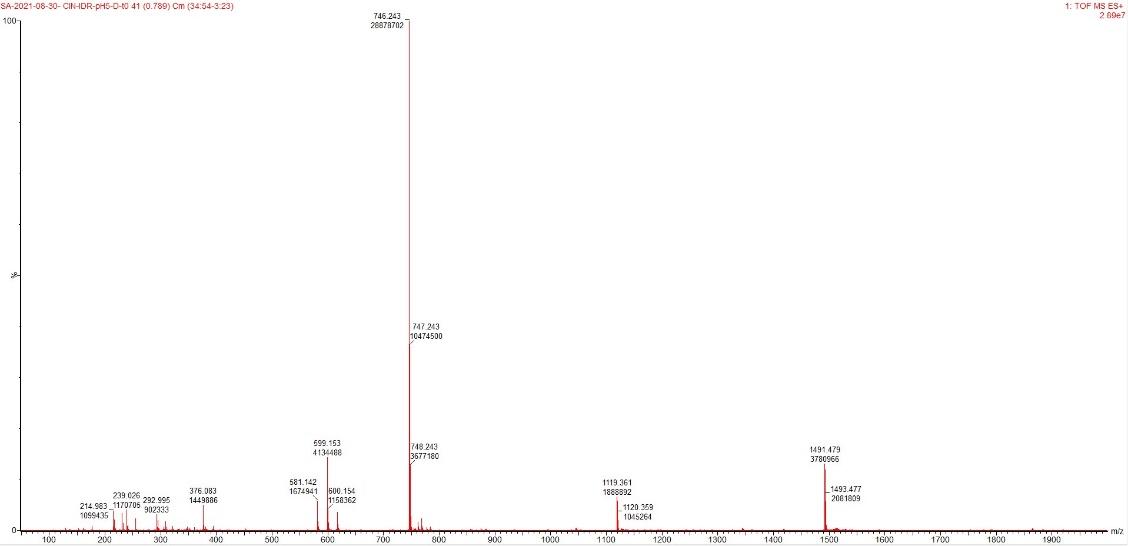


**Figure S6**. MS spectrum of the proDoxo after purification by digestion; Doxo [M-H^+^] 544.1346 and proDoxo [M-H^+^] 746.243.

**
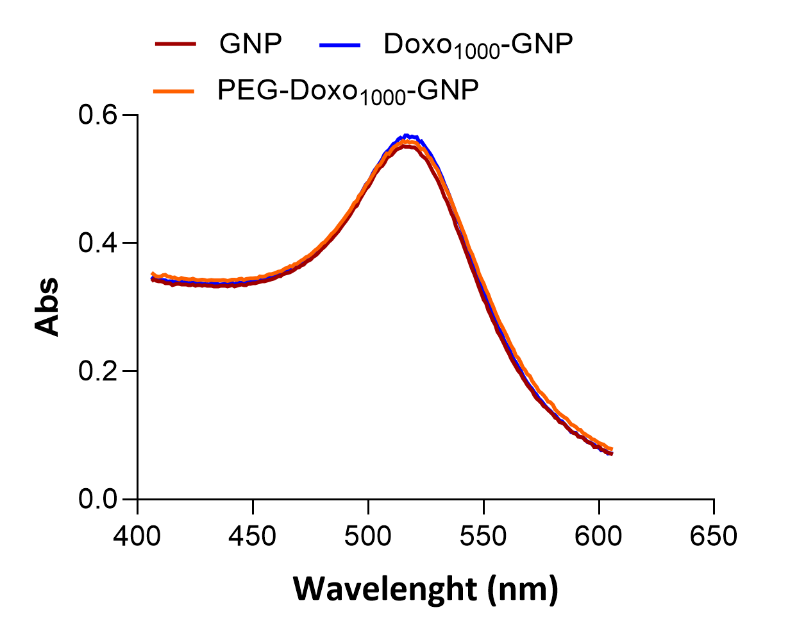
**

**Figure S7.** UV-Vis spectra of GNPs, and representative Doxo loaded GNPs, and PEGylated Doxo loaded GNPs.

| 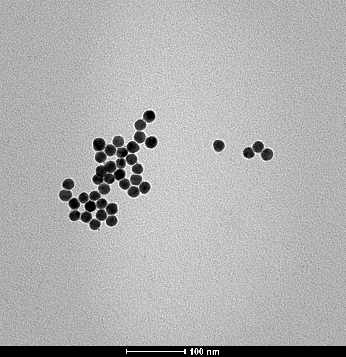 A | 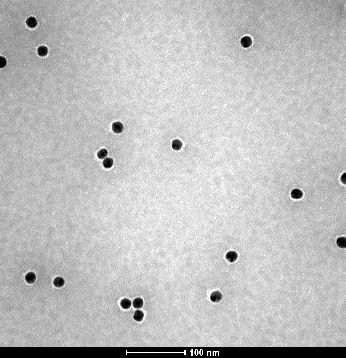 B | 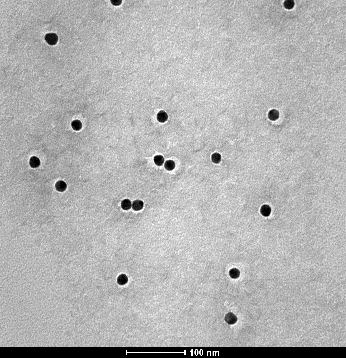 C | 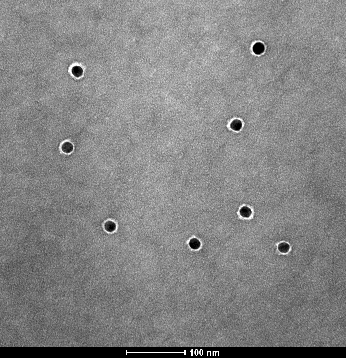 D |
| --- | --- | --- | --- |
|  | 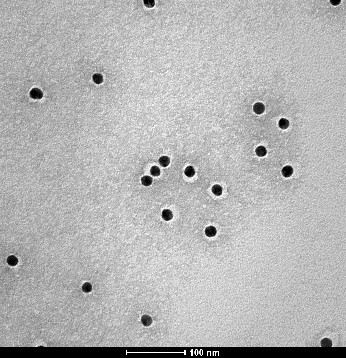 |  |  |
|  | E | F 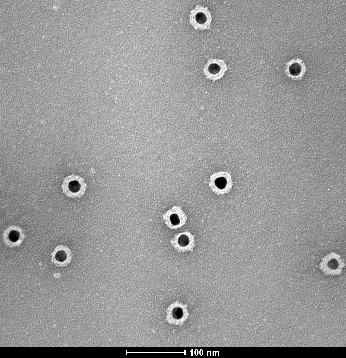 | G 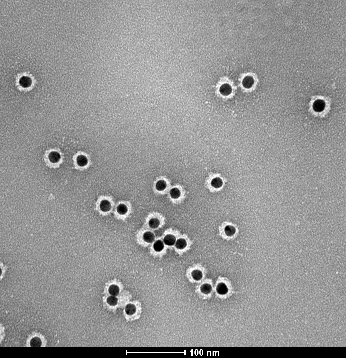 |

**Figure S8**. TEM images of GNP decorated at increasing proDoxo/(FA-BDP)-GNP molar ratios. (A) Freshly prepared GNP, (B) FA-PEG-Doxo_0_-GNP , (C) FA-PEG-Doxo_200_-GNP , (D) FA-PEG-Doxo_500_-GNP, (E) FA-PEG-Doxo_700_-GNP, (F) FA-PEG-Doxo_1000_-GNP and (G) FA-PEG-Doxo_1500_-GNP. Samples were negatively stained with uranyl acetate. Scale bars on panels: 100 nm.

| 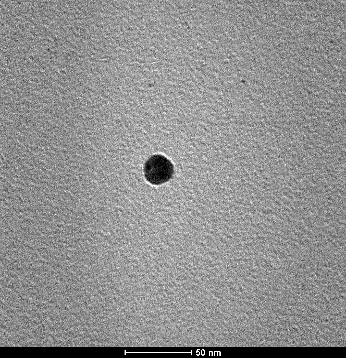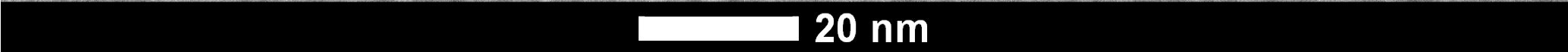 A | 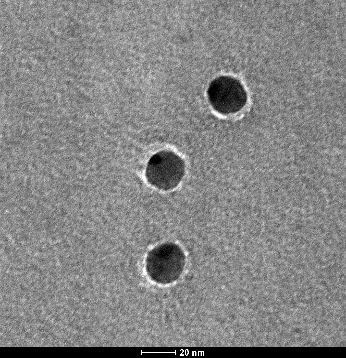 B | 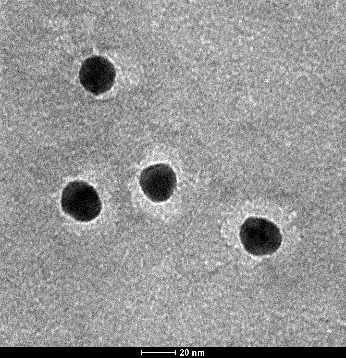 C | 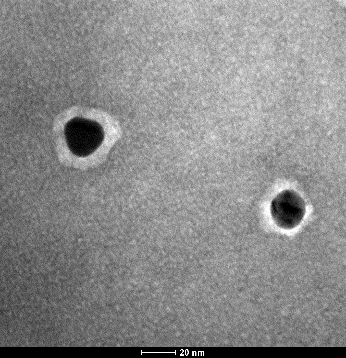 D |
| --- | --- | --- | --- |
|  |  |  |  |
|  | E 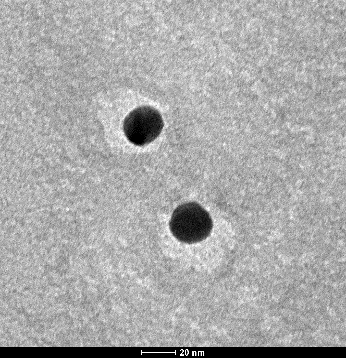 | F 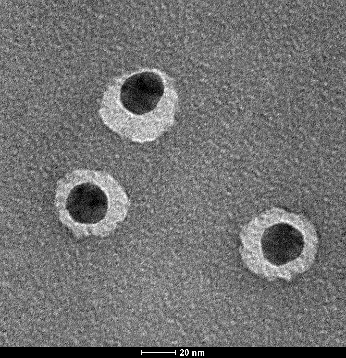 | G 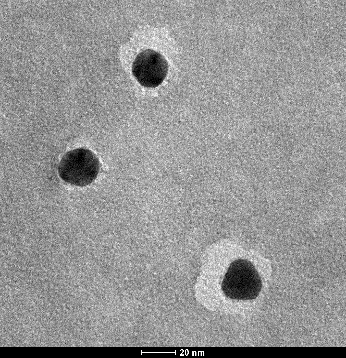 |

**Figure S9**. TEM images of GNP decorated at increasing proDoxo/FA-GNP molar ratios. (A) Freshly prepared GNP, (B) FA-PEG-Doxo_0_-GNP, (C) FA-PEG-Doxo_200_-GNP, (D) FA-PEG-Doxo_500_-GNP, (E) FA-PEG-Doxo_700_-GNP, (F) FA-PEG-Doxo_1000_-GNP, (G) FA-PEG-Doxo_1500_-GNP . Coated GNP were negatively stained with uranyl acetate. Scale bars on panels: 20 nm.


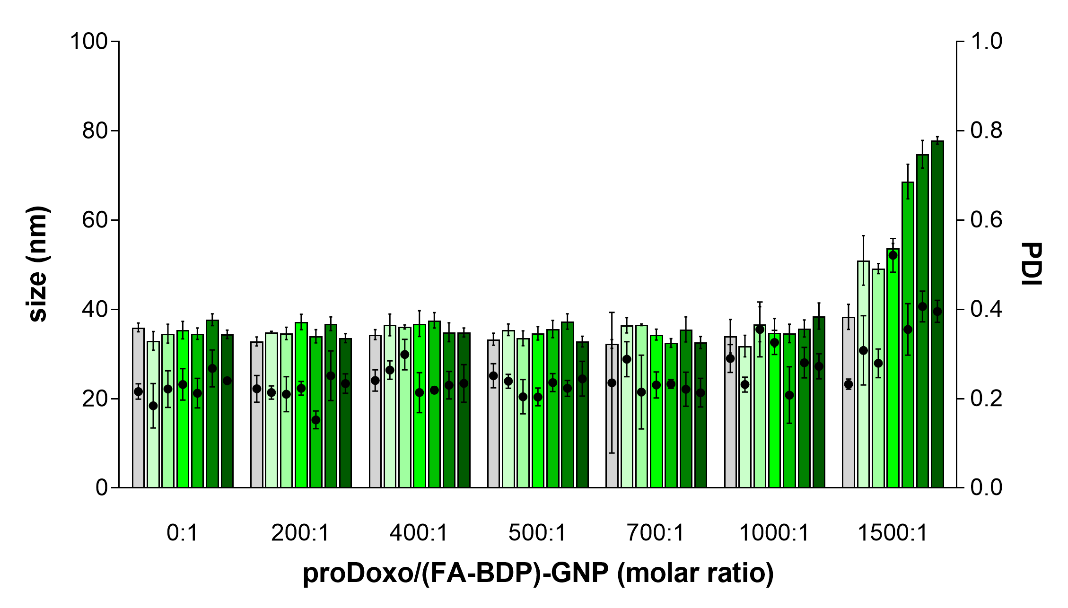


**Figure S10**. Colloidal stability of FA-PEG-Doxo-GNP in protein-containing medium. Size profile over time of formulations decorated with increasing proDoxo/FA-GNP molar ratios in FF-DMEM medium added of 10 % FBS at time 0 h (■) and after 2 (■), 4 (■), 6 (■), 24 (■), 48 (■), 72 (■) h at 37 °C. Particles were analyzed by DLS. Polydispersity Index (PDI, ●) refers to the right axis. Size is reported in Intensity.


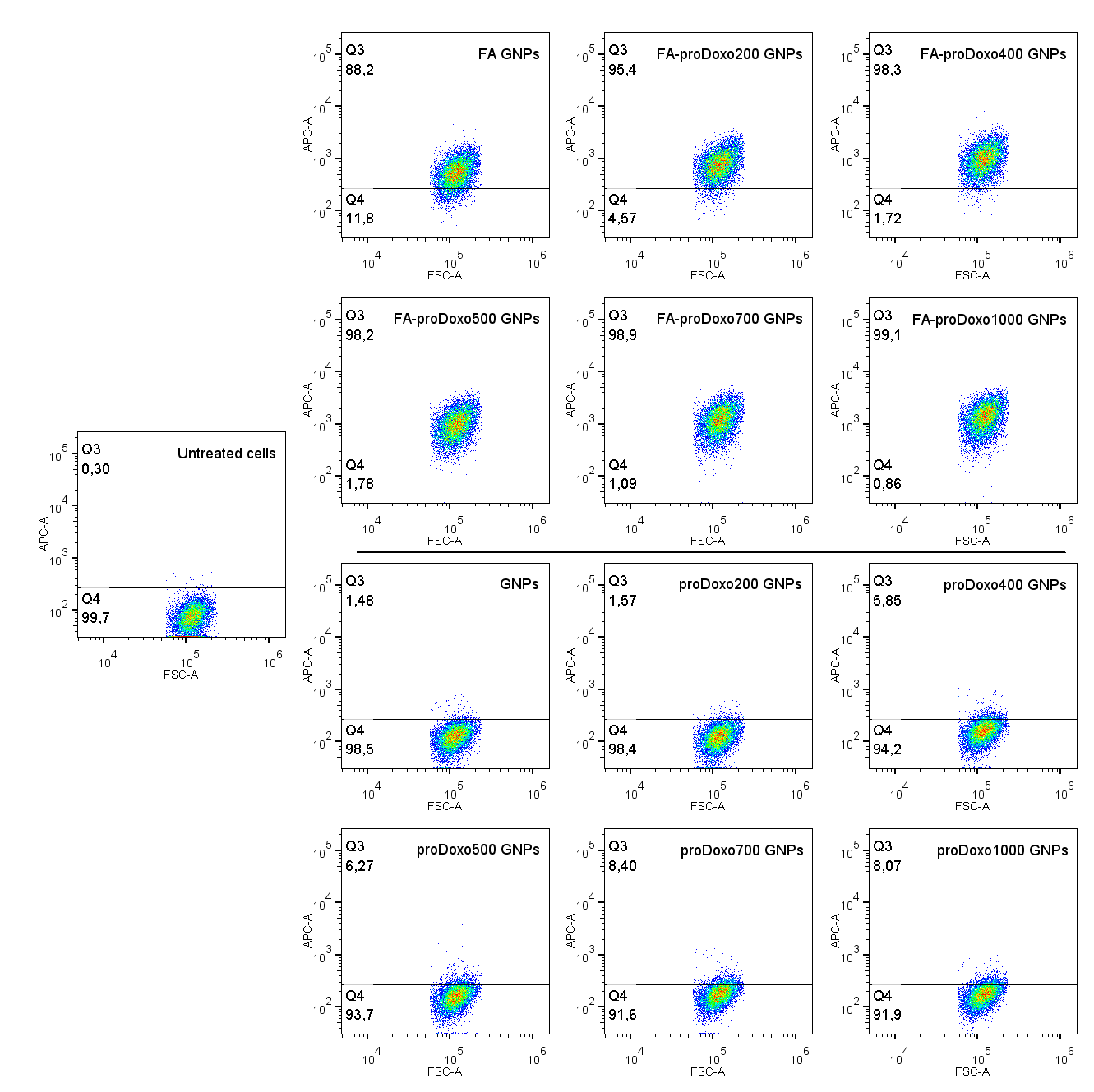


**PEG-Doxo_700_-(BDP)**

**GNP**

**PEG-Doxo_500_-(BDP)**

**GNP**

**PEG-Doxo_400_-(BDP)**

**GNP**

**PEG-(BDP)**

**GNP**

**PEG-Doxo_1000_-(FA-BDP)**

**GNP**

**PEG-Doxo_500_-(FA-BDP)**

**GNP**

**PEG-Doxo_700_-(FA-BDP)**

**GNP**

**PEG-Doxo_400_-(FA-BDP)**

**GNP**

**PEG-Doxo_200_-(FA-BDP)**

**GNP**

**PEG-Doxo_1000_-(BDP)**

**GNP**

**PEG-Doxo_200_-(BDP)**

**GNP**

**PEG-(FA-BDP)-GNP**

**Untreated cells**

**Figure S11**. Flow cytometric profiles of KB^FR+^ cells incubated for 6 hours with targeted BDP labelled FA-PEG-Doxo-GNP or untargeted PEG-Doxo-GNP prepared at increasing proDoxo/FA-GNP molar ratios. Cells positives for BDP fluorescence are gated in Q3.

**
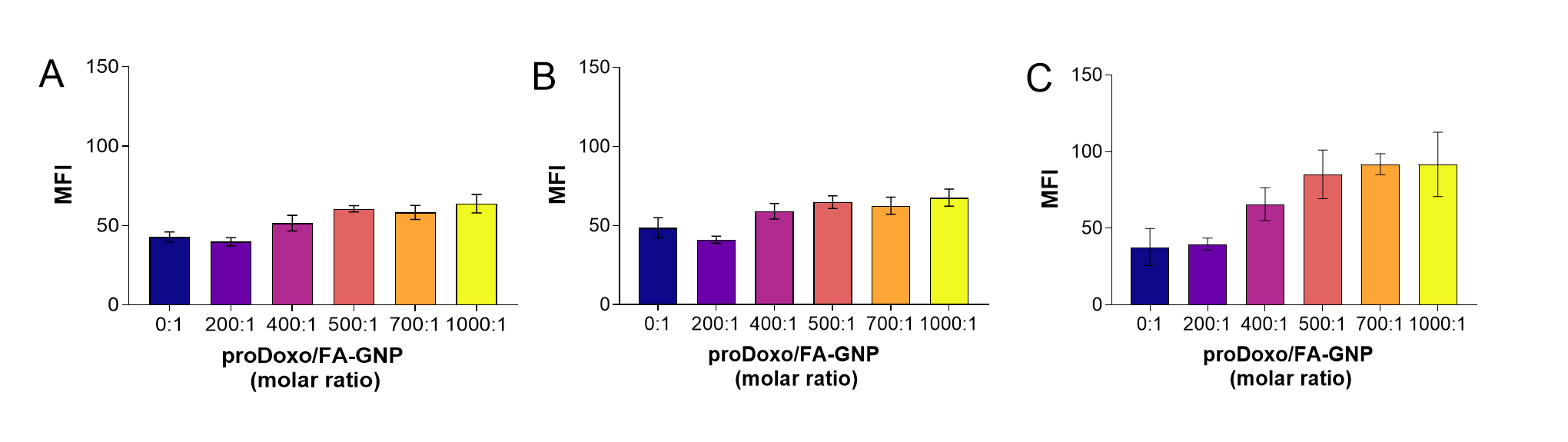
**

**Figure S12**. Cellular association profiles of KB^FR+^ cells incubated with untargeted (PEG-Doxo-GNP) nanoparticles for (A) 2, (B) 4, or (C) 6 h. GNP were decorated with increasing proDoxo/FA-GNP molar ratios. Results are reported as means of three independent experiments ± standard deviations. MFI values refer to BDP fluorescence. Figure displays the same results reported in Figure 4 for untargeted GNP with a narrower range of y axes.


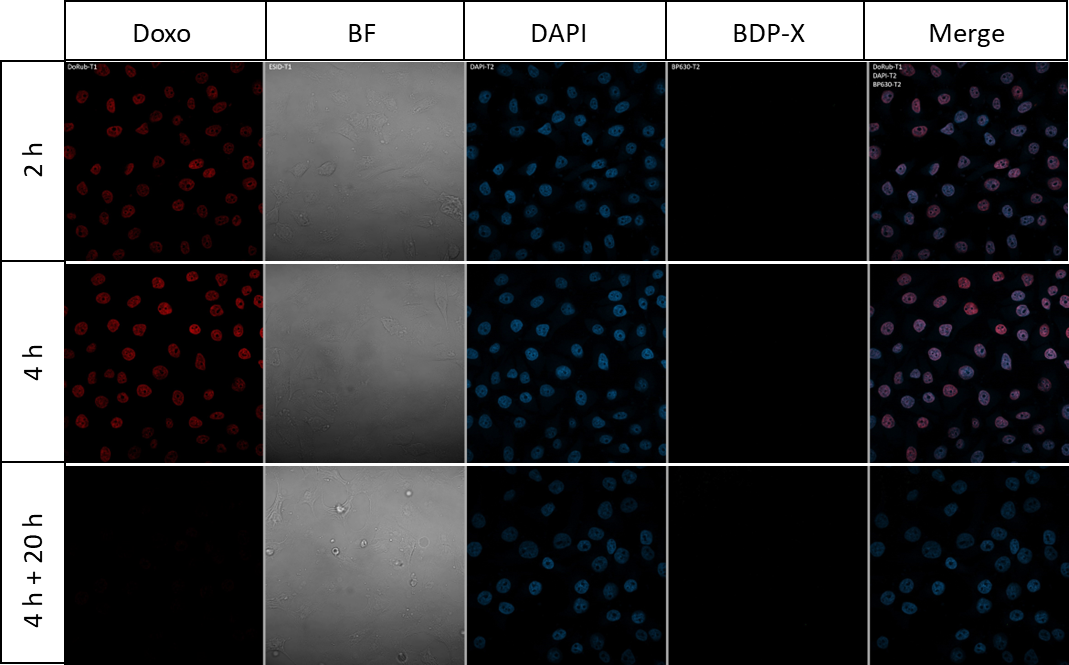


10 µm

10 µm

10 µm

10 µm

10 µm

10 µm

10 µm

10 µm

10 µm

10 µm

10 µm

10 µm

10 µm

10 µm

10 µm

Brightfield

BDP

**Figure S13**. Confocal laser microscopic images of KB^FR+^ cells incubated with free Doxo for 2, or 4 h, or for 4 h followed by washing and further growth for 20 hours in fresh medium. The concentration of Doxo in medium was 5 μM. The cells were stained with DAPI (light blue) for nuclei; Doxo fluorescence is displayed in red. The microscope was equipped with a 40x objective. Scale bars on panels: 10 µm.

**References**

[1] Brazzale C, Mastrotto F, Moody P, et al. Control of targeting ligand display by pH-responsive polymers on gold nanoparticles mediates selective entry into cancer cells. Nanoscale. 2017;9(31):11137-11147.

[2] Brazzale C, Canaparo R, Racca L, et al. Enhanced selective sonosensitizing efficacy of ultrasound-based anticancer treatment by targeted gold nanoparticles. Nanomedicine (London, England). 2016;11(23):3053-3070.

[3] Zhang K, Rossin R, Hagooly A, et al. Folate-mediated Cell Uptake of Shell-crosslinked Spheres and Cylinders. Journal of polymer science Part A, Polymer chemistry. 2008;46(22):7578-7583.

[4] Caliceti P, Salmaso S, Semenzato A, et al. Synthesis and physicochemical characterization of folate-cyclodextrin bioconjugate for active drug delivery. Bioconjugate chemistry. 2003;14(5):899-908.

[5] Sims GE, Snape TJ. A method for the estimation of polyethylene glycol in plasma protein fractions. Analytical biochemistry. 1980;107(1):60-3.

[6] Turkevich J, Stevenson PC, Hillier J. The Formation of Colloidal Gold. The Journal of Physical Chemistry. 1953;57(7):670-673.

[7] Liu X, Atwater M, Wang J, et al. Extinction coefficient of gold nanoparticles with different sizes and different capping ligands. Colloids and surfaces B, Biointerfaces. 2007;58(1):3-7.

[8] Jain PK, Lee KS, El-Sayed IH, et al. Calculated absorption and scattering properties of gold nanoparticles of different size, shape, and composition: applications in biological imaging and biomedicine. The journal of physical chemistry B. 2006;110(14):7238-48.

[9] Link S, El-Sayed MA. Spectral Properties and Relaxation Dynamics of Surface Plasmon Electronic Oscillations in Gold and Silver Nanodots and Nanorods. The Journal of Physical Chemistry B. 1999;103(40):8410-8426.
